# Emerging Human Fascioliasis in India: Review of Case Reports, Climate Change Impact, and Geo-Historical Correlation Defining Areas and Seasons of High Infection Risk

**DOI:** 10.3390/tropicalmed10050123

**Published:** 2025-05-02

**Authors:** Santiago Mas-Coma, Pablo F. Cuervo, Purna Bahadur Chetri, Timir Tripathi, Albis Francesco Gabrielli, M. Dolores Bargues

**Affiliations:** 1 Departamento de Parasitología, Facultad de Farmacia, Universidad de Valencia, Av. Vicente Andrés Estellés s/n, Burjassot, 46100 Valencia, Spain; pablo.f.cuervo@uv.es (P.F.C.); m.d.bargues@uv.es (M.D.B.); 2 CIBER de Enfermedades Infecciosas, Instituto de Salud Carlos III, C/Monforte de Lemos 3-5, Pabellón 11, Planta 0, 28029 Madrid, Spain; 3 Department of Biotechnology-North East Centre for Agricultural Biotechnology, Assam Agricultural University, Jorhat 785013, India; purna.chetri@aau.ac.in; 4 Molecular and Structural Biophysics Laboratory, Department of Zoology, North-Eastern Hill University, Shillong 793022, India; timir.tripathi@gmail.com; 5 Global Neglected Tropical Diseases Programme (NTD), World Health Organization, Avenue Appia 20, CH-1211 Geneva, Switzerland; gabriellia@who.int

**Keywords:** *Fasciola gigantica*, *F. hepatica*, case report review, climate change analyses, disease transmission, geo-historical assessments, risky areas, epidemiological scenario, human infection risk, India

## Abstract

The trematodes *Fasciola hepatica* and *F. gigantica* are transmitted by lymnaeid snails and cause fascioliasis in livestock and humans. Human infection is emerging in southern and southeastern Asia. In India, the number of case reports has increased since 1993. This multidisciplinary study analyzes the epidemiological scenario of human infection. The study reviews the total of 55 fascioliasis patients, their characteristics, and geographical distribution. Causes underlying this emergence are assessed by analyzing (i) the climate change suffered by India based on 40-year-data from meteorological stations, and (ii) the geographical fascioliasis hotspots according to archeological–historical records about thousands of years of pack animal movements. The review suggests frequent misdiagnosis of the wide lowland-distributed *F. gigantica* with *F. hepatica* and emphasizes the need to obtain anamnesic information about the locality of residence and the infection source. Prevalence appears to be higher in females and in the 30–40-year age group. The time elapsed between symptom onset and diagnosis varied from 10 days to 5 years (mean 9.2 months). Infection was diagnosed by egg finding (in 12 cases), adult finding (28), serology (3), and clinics and image techniques (12). Climate diagrams and the Wb-bs forecast index show higher temperatures favoring the warm condition-preferring main snail vector *Radix luteola* and a precipitation increase due to fewer rainy days but more days of extreme rainfall, leading to increasing surface water availability and favoring fascioliasis transmission. Climate trends indicate a risk of future increasing fascioliasis emergence, including a seasonal infection risk from June–July to October–November. Geographical zones of high human infection risk defined by archeological–historical analyses concern: (i) the Indo-Gangetic Plains and corridors used by the old Grand Trunk Road and Daksinapatha Road, (ii) northern mountainous areas by connections with the Silk Road and Tea-Horse Road, and (iii) the hinterlands of western and eastern seaport cities involved in the past Maritime Silk Road. Routes and nodes are illustrated, all transhumant–nomadic–pastoralist groups are detailed, and livestock prevalences per state are given. A baseline defining areas and seasons of high infection risk is established for the first time in India. This is henceforth expected to be helpful for physicians, prevention measures, control initiatives, and recommendations for health administration officers.

## 1. Introduction

Fascioliasis is caused by liver flukes of the trematode genus *Fasciola* in infections of humans and herbivorous animals, including ruminants (mainly sheep, cattle, goats, and buffaloes), equines (mainly donkeys and mules, less frequently horses), and camelids of the Old World (two-humped camel and one-humped dromedary), but also omnivores such as pigs and wild boars [1]. Three species are recognized within the genus *Fasciola*. Experimental studies and extensive field surveys have confirmed that the species *F. nyanzae* is specific to the hippopotamus [2]. On the contrary, the two other species are less host specific and include *F. hepatica* in Europe, Asia, Africa, the Americas, and Oceania, and *F. gigantica*, which is restricted to parts of Asia and Africa [1].

In recent decades, fascioliasis has proved to be of public health importance not only in the veterinary field [3] but also in medicine because of the disease it causes in humans [4]. The World Health Organization has therefore included fascioliasis within the list of neglected tropical diseases [5,6] by considering its pathogenicity, which is higher in the infection by the larger *F. gigantica* than by the smaller *F. hepatica* adult stage [7], including severe neurological and ocular manifestations [8], long-term sequelae even in some treated patients [9], and underdevelopment consequences in children and rural communities [10].

*Fasciola* species share the same two-host life cycle pattern, including the definitive mammal host and the molluscan vector belonging to taxa of amphibious snails of the family Lymnaeidae. Fascioliasis epidemiologically behaves similarly to arthropod-borne diseases and justifies the use of the term “vector” for the lymnaeid snail transmitters. As in all vector-borne diseases, the specificity of the parasite regarding the vector species defines the geographical distribution of the disease. In fascioliasis, the existence of lymnaeid species of the *Galba*/*Fossaria* group throughout all continents explains the worldwide distribution of *F. hepatica*, whereas the restriction of lymnaeid species of the *Radix* group to Asia and Africa underlies the existence of *F. gigantica* in only these two continents [1,11].

This life cycle comprises four phases [12], which depend on environmental factors and human activities influencing transmission rates and consequent modifications in disease prevalences, intensities, and potential geographical spread:

- Adult stage and egg shedding: The definitive host is infected by ingestion of metacercariae [13]. After excystment, juvenile flukes migrate from the intestine to the liver, where they develop into adult flukes, which attain sexual maturity in 3–4 months inside biliary canals and/or the gallbladder (invasive, migratory, or acute phase), following a life span of between 9 and 13.5 years (biliary, obstructive, or chronic phase), during which adults produce eggs that reach the external milieu by way of the bile, intestines, and stool. This phase is crucial for the maintenance of prevalences and intensities of the disease in an endemic area [14,15], and for its wide intercontinental geographical spread, as occurred with human-guided movements of pack animals for thousands of years from the Neolithic Age [1], and as recently occurred due to livestock management in the countrywide spread of human fascioliasis in Vietnam without any influence of climate change [16].

An additional key aspect in the geographical spread of fascioliasis is the capacity of lymnaeid snails to be passively transported by livestock. Most lymnaeids able to transmit fascioliasis show pronounced amphibious behavior, which leads them to be widely present in the mud surrounding the freshwater collections to which livestock go for drinking. Lymnaeids may thereby be transported from one place to another by remaining on the mud attached to the hooves of the animals. This, together with the capacity of lymnaeids to hibernate and estivate on dry mud, explains how fascioliasis spreads over great distances by taking advantage of the numerous caravans of pack animals used along the great routes followed for thousands of years in the past in Asia and Africa, as well as across the ocean with old ships to colonize the Americas and Oceania [1].

Recent and present exportation/importation of livestock between countries of different continents is adding new problems of hybridization between *F. hepatica* and *F. gigantica*, mainly in Asia [17,18].

The other three phases of the life cycle develop in the freshwater milieu [12], which means that fasciolid transmission is only possible where surface freshwater collections inhabited by populations of susceptible lymnaeid snail species are available:

- Egg and miracidium: The transition between definitive mammal host and intermediate snail host includes the long resistance phase of the egg and the short active phase of the miracidium. The fluke eggs shed with the mammalian feces will continue their development in freshwater of appropriate physicochemical characteristics.
- Intramolluscan development and cercariae shedding: The development at snail level includes miracidium penetration, the sporocyst stage, redial generation, the production of cercariae, and shedding of the latter into water.
- Cercariae and metacercariae: The transition between snail and mammal hosts includes the short swimming phase of cercariae and the long resistance phase of metacercariae. Cercariae are shed by the snail and swim for a short time until contacting a solid support, mostly leaves of water plants above or below the water line, to attach and give rise to the metacercarial cyst, which becomes infective within 24 h.

Climate change is a global increasing phenomenon able to alter the prevalences, intensities, and geographical distribution of fascioliasis because of the many climate factors influencing liver fluke transmission rates, such as temperatures and global warming, rainfall and humidity leading to surface water availability, and sunshine and evapotranspiration [19]. Two studies using fascioliasis risk indices, which rely on climate factors influencing liver fluke transmission, have for the first time demonstrated significant links between climate change and human fascioliasis. In the Pakistani Punjab, climate and anthropogenic environmental modifications influence both geographical distribution and the seasonality of fascioliasis risks. The local emergence of human fascioliasis proved to be the consequence of an increasing infection risk when the original transmission mono-seasonality peak marked by the increasing rainfall within the monsoon’s climate has given rise to a bi-seasonality, including a second transmission peak related to water management throughout a very wide 60,000 km-long artificial canal irrigation system [20]. Recently, the global warming phenomenon has proven to be the cause of the geographical spread of human fascioliasis infection risk northward, southward, and to higher altitudes surrounding the human fascioliasis hyperendemic area of the Bolivian Altiplano [21].

Regarding the current geographical distribution of fascioliasis, we know today that routes used for the human-guided movements of pack animals during past epochs of thousands of years transporting fasciolid flukes in the liver and fecally shedding their eggs, as well as lymnaeid snail vectors in mud attached to the hooves of the animals, gave rise to fascioliasis transmission that strongly adhered to the areas through which those routes were running [1]. Such areas where fascioliasis thereby reached high transmission rates have been observed to be linked to higher human infection risk. Hence, this type of analytical approach has already proven its usefulness and capacity to understand the present distribution of this disease in different fascioliasis-endemic areas.

The present study aims to review the increasing number of fascioliasis patients reported in India, to analyze their characteristics, and to assess the causes underlying the emergence of this disease. Our analysis focuses on the geographical distribution of these reports, and a multidisciplinary attempt to define a baseline for future research and assess this emergence. We analyze the potential cause–effect relationships of the emergence with (i) the climate change suffered by India in last decades, and (ii) the geographical hotspots of fascioliasis suggested by the archeological and historical records about the thousands of years of pack animal caravans. These two analytical studies furnish a baseline defining geographical areas and seasonal periods of human infection risk. This new baseline is henceforth expected to be helpful for individual prevention methods and general control initiatives.

## 2. Materials and Methods

### 2.1. Literature Search for Human Infection Reports

A wide literature review to find reports of patients infected with *Fasciola* was performed by analyzing databases, web entries, web platforms, multititle packages, libraries, free collections, and personal e-mail requests when appropriate. The key criteria used to select the papers were the contents on (i) human infection by *F. hepatica* or *F. gigantica*, and (ii) patients diagnosed in India and/or Indian citizens. No restriction was applied to the period of time taken into consideration, i.e., from the beginning of the oldest publication on human infection up to the present.

Keywords for literature searches included combinations of the following terms: fasciol, fluke, trematode, digenean, liver, hepatic, human, patient, diagnos, serol, coprol, stool, ultrasound, age, years, clinic, symptom, patho, eosinophil, anamnesis, epidemi, hospital, treatment, and fasciolicide. These terms were used in English, although other languages were not excluded when interesting information was found.

Reviews and books were in need to be read in full because the appropriate information did not appear in titles, abstracts, summaries, or keywords. Literature noted as references in published reports was useful. International library networks were used to obtain publications not appearing on the internet or in cases in which only the title and/or the abstract could be found in internet databases, such as, for instance, the Helminthological Abstracts of CABI, PubMed, and others.

Information on unpublished human infection patients diagnosed in India were obtained from the World Health Organization (WHO) in cases in which the physicians contacted the WHO Program for Triclabendazole Donation [22] for the request of Egaten^®^ (triclabendazole for human treatment). It should be considered that, among the strategies that WHO coordinates to enhance activities of prevention and control of neglected tropical diseases, the WHO and Novartis Pharma AG (Basel, Switzerland) reached an agreement whereby Novartis donates Egaten^®^ (oral tablets of 250 mg triclabendazole for human use) for fascioliasis treatment in endemic countries. This process of donation to the WHO started in 2005, and the WHO began to distribute this medicine to endemic countries starting in 2006. Instructions for endemic countries to apply for donated triclabendazole are available on the WHO’s website [22].

### 2.2. Review and Quality Assessment

No restrictions were applied to sources, i.e., searches were not restricted to medical sources. Special efforts were dedicated to obtaining copies of reports not available in electronic databases. The main criteria used to select the reports included verification that infection by *Fasciola* spp. was not misdiagnosed and that the information provided was sufficiently detailed. In several cases, the shortness of the reports underlies an insufficiency of crucial aspects in the diagnosis of patients.

Moreover, in India, the consideration of fascioliasis as a disease of secondary importance probably led to the highly scattered diffusion of the reports in many kinds of journals of pronouncedly different coverages concerning health fields, hospital activities, postgraduate medicine, science, and research. This scattering explains the difficulties in obtaining human fascioliasis reports in India by using current digital search tools. This is why authors of reports always note that only a very few human infection cases have been reported in India. This evidence led us to deeply examine each report. The main problematic aspects faced during search tasks are appropriately analyzed in the Section 4.

In this way, a total of 59 reports of individual human fascioliasis cases, among which 4 reports concerned 4 duplicate patients (i.e., the same patient already having been reported), was found in a total of 35 published articles [18,23,24,25,26,27,28,29,30,31,32,33,34,35,36,37,38,39,40,41,42,43,44,45,46,47,48,49,50,51,52,53,54,55,56] and 3 unpublished reports of 3 patients having contacted the WHO to obtain Egaten^®^ for their treatment.

### 2.3. Climate Analyses

**Figure 1:**
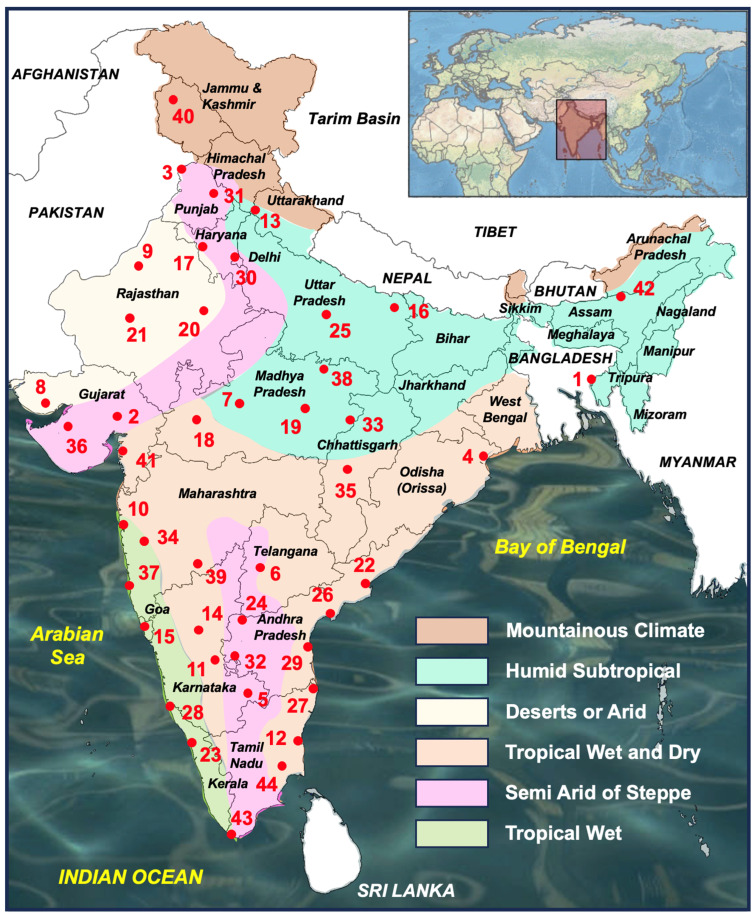
Location of the 44 meteorological stations analyzed on the map of India showing the distribution of climate zones according to states and union territories of the country. Borders of climate zones concern yearly averages. Meteorological stations are detailed in Table 1. Original by P.F. Cuervo and S. Mas-Coma.

Daily temperature and precipitation data concerning a 40-year period (1981–2020) were obtained from 44 meteorological stations distributed throughout India. The required meteorological data were obtained from the National Centers for Environmental Information of the United States of America (https://www.ncdc.noaa.gov/cdo-web/, accessed on 24 October 2024). Details on the location of the meteorological stations used are included in Figure 1 and Table 1. These daily data were processed to obtain the monthly values of the climatic factors required for the calculation of fascioliasis climatic risk indices.

Monthly climatic data calculated from the daily data included (see the Abbreviations section, below) MET, MMT, MmT, EMT, EmT, MTD (i.e., MMT–MmT), ETD (i.e., EMT–EmT), all in °C; prcp in mm; and DP (i.e., prcp ≥1), DF (i.e., t_min_ < 0 °C), and PET in mm.

### 2.4. Checking and Completion of Missing Data

**Figure 2:**
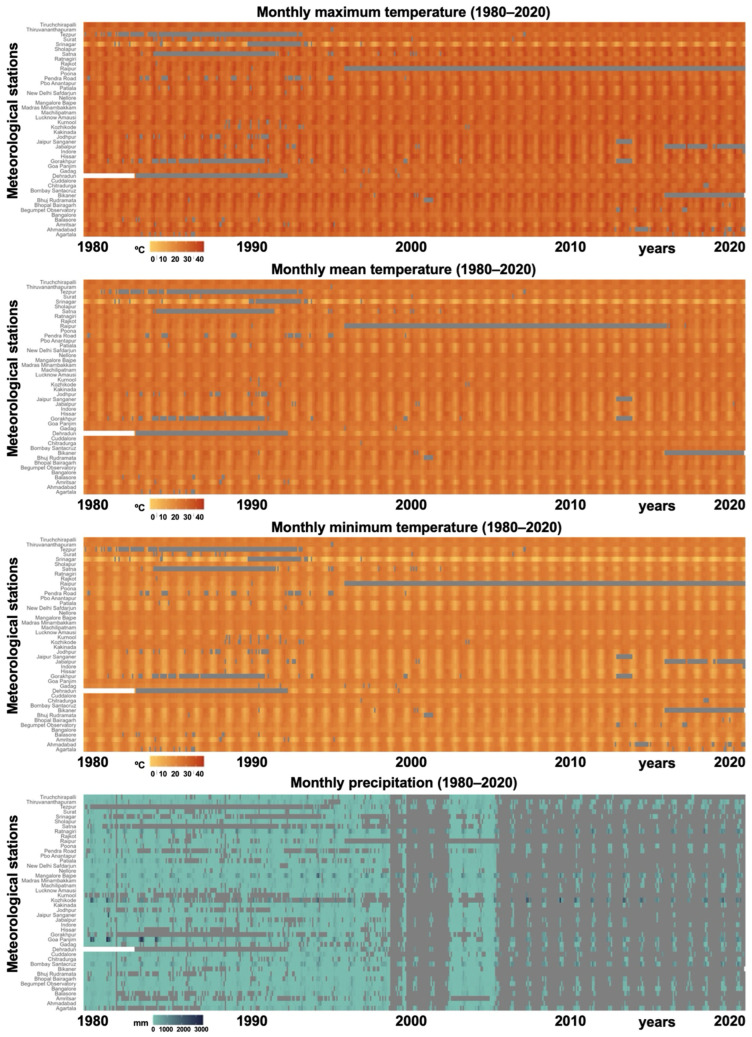
Heatmaps indicating the monthly data availability for the main climatic factors (maximum temperature, mean temperature, minimum temperature, and precipitation) relevant for the calculation of the fascioliasis forecast indices for the 1980–2020 period, according to the meteorological stations. The color ramps indicate the magnitude of the climatic factor, and the gray color indicates the absence of data. For names of meteorological stations, see the 44 numbers in Table 1 (use magnifying zoom to see the numbers better if needed). Original by P.F. Cuervo.

The percentage of monthly climatic data available is indicated in Table 1. The availability of data per main climatic factor (temperature and precipitation) was verified by plotting on a heatmap the monthly values for each meteorological station and for the entire period under study (1980–2020) (Figure 2). The lack of precipitation data in the last couple of decades is evident.

In order to complete the missing precipitation data, monthly precipitation data for the period 1980–2020 was downloaded from three additional climatological databases, based on either remote sensing data (GPCP [57]) or interpolation modeling from land-based meteorological stations (CHELSA Climate v2.1 [58]; CRU TS v4.08 [59]). Monthly precipitation time series were extracted for each meteorological station from each of these sources. After extraction, the Spearman’s correlation between these new time series and the original precipitation data was assessed to select the one that fit better. The time series with the best correlation was used to replace the original precipitation data and later implemented in the calculation of the climatic forecast indices.

### 2.5. Climatic Forecast Indices

The incidence of fascioliasis infection in the definitive host is related to air temperature, rainfall, and/or potential evapotranspiration [60]. The two most useful indices have proven to be the Wet Day index or Mt index [61], which was later improved [62,63], and the Water budget-based system index or Wb-bs index [64], which was later modified for large-scale regional use [65]. Given that the Mt index does not differentiate between *F. hepatica* and *F. gigantica*, we restricted our analyses to the use of the Wb-bs index.

The Water budget-based system (Wb-bs), adapted for large-scale regional use with monthly climate data, was calculated following the original equation below [65]:

Wb-bs = (*GDD* × days in month), if [*R* − (*PET* 0.8)] > 0,

+ (*GDD* × *z*)[(*R* − *PET*)/25], if (*R* − *PET*) > 0

where *R* is the rainfall, *PET* the potential evapotranspiration, *z* the number of surplus rainy days in the month (calculated as the mode for each particular station), and *GDD* is the growing degree-days, calculated as the monthly MET minus the base development temperature for *F. hepatica*, which is 10 °C [61,66], or for *F. gigantica*, which is 16 °C [67].

In the first part of the formula, subtracting the factor (*PET* × 0.8) from rainfall (*R*) is equivalent to counting monthly *GDD* if rain-dependent moisture storage is present in the top 2.5 cm layer of a soil water budget model [65]. The second part counts GDD if monthly surplus water is present due to rainfall events [65]. Risk values conventionally established, and later used by several authors, are: 600 = no risk, 601–1500 = low risk, 1500–3000 = moderate risk, and 3000 = high risk [19,20,68,69].

For every climatic forecast index, *PET* was calculated according to the method originally proposed [70] and later modified [71]. This method was preferred since it requires minimal climatic data (temperature and solar radiation), and most meteorological stations only record temperature and rainfall. Its results are closely correlated with those obtained by the frequently proposed Penman method [72,73] and has already been used for the estimation of *PET* and the calculation of climatic forecast indices [16,21,74].

Meteorological stations to be analyzed were selected in consideration of the geographical distribution of the human fascioliasis cases reported. Indices were calculated for each month/year, producing a data set from which the following was obtained for each station: (i) monthly values for the 1980–2020 period, and (ii) monthly means for 1980–2020.

Climate and seasonality at each of these meteorological stations was assessed by representing mean monthly data of the climate factors in climate diagrams. Climate diagrams illustrate the year-round profile of monthly average values for temperature and precipitation, providing a brief summary of average climatic conditions. Climate diagrams were produced for each meteorological station considered using a modified script from the R package climatol (https://climatol.eu/ accessed on 24 October 2024). Roughly, when the precipitation curve undercuts the temperature curve, the area in between them indicates the dry season, whereas when the precipitation curve supersedes the temperature, the curve indicates the moist season.

### 2.6. Spatial and Statistical Analyses

All the necessary calculations, spatial analyses, and statistics applied for the methods detailed in the aforementioned three sections of climate analyses, checking and completion of missing data, and climate forecast indices were carried out with R statistical software (“R: A language and environment for statistical computing,” version 4.2.2 [31 October 2022 ucrt], http://www.r-project.org) and RStudio 2022.02.3.492 (“RStudio: Integrated development environment for R,” http://www.rstudio.com/ accessed on 24 October 2024).

### 2.7. Geo-Historical Analyses

Studies suggest that the repeated round-trips of back-and-forth movements of very numerous animal hosts infected by fasciolids and passively transporting lymnaeid vector species may have led to the genetic enrichment of both fasciolids and lymnaeids by evolutionary repeated crossbreeding. Although fasciolids are hermaphroditic trematodes, we know today that cross-fecundation at the fasciolid adult stage within the same individual liver is more usual than previously thought. Similarly, lymnaeid species may fecundate by selfing, but field and experimental observations prove that these freshwater snails use to go for cross-fecundation immediately after arriving to a new milieu and environment.

The thousands of years of caravans of pack animals furnished a sufficiently long period as to allow both the free-living larval stages of fasciolids and lymnaeid snails to adapt to the local characteristics of the milieu, the environment, and the climatic conditions. Both fasciolid transmission and the presence of lymnaeid populations are known to have the capacity to go on with their existence in a given place after hundreds of years [1]. Repeated backcrossing with the parental taxa leads to an increase in the fitness of the offspring, which underlies a timely progressive adaptation to a local environment. Such a local adaptation is crucial for the long-term maintenance of the life cycle dynamics of *Fasciola* species, unless the local place is destroyed or past freshwater collections disappear.

The aforementioned facts apply for past long roads and routes repeatedly followed by transhumant herders, nomadic ethnic groups and their livestock, and pastoralists locally moving their herds around the same pasture fields, and, of course, for farmers keeping their animals within fenced areas. It should be considered that anti-fasciolid drugs were not known or administered in the past epochs, nor was the existence and pathogenicity of the hepatic fasciolid flukes infecting livestock known [1].

With this baseline in mind, we looked for a potential overlap between the geographical distribution of the fascioliasis patients and the past scenarios of the old routes and respective nodes by combining archeological and historical records of India [1], and in data furnished by governmental offices of the Indian states and available in digital sources. Search efforts for specific information focused on each one of the Indian states, covering from past times from the end of the Neolithic period, i.e., several thousands of years BC, until the present situation.

## 3. Results

### 3.1. Review of Case Reports

**Figure 3:**
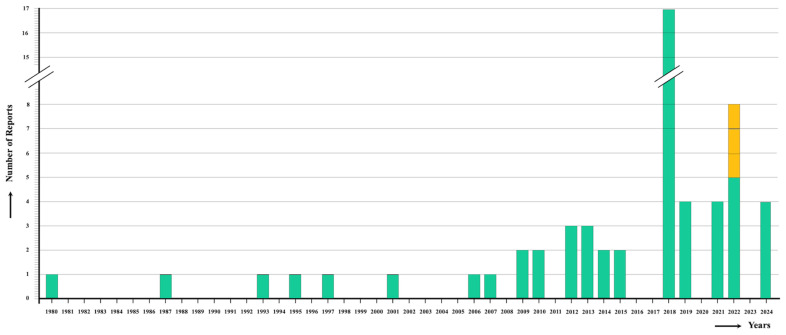
Graph showing the increasing chronological accumulation of human fascioliasis case reports in India according to year. Green: published reports; orange: unpublished cases reported to the WHO for specific treatment request. Original by S. Mas-Coma.

The review identified a total of 59 reports (Table 2). The chronological analysis of the reports shows an evident emergence from the year 2006 (Figure 3). These 59 reports indeed concern 55 human individual cases, because four reports concern given human infections that were already included in previous articles, i.e., the infection of the same patients analyzed from other points of view or with different methods and techniques.

Among these 55 cases, the locality of residence is only noted in 11 patients, whereas for the remaining 44 patients only the locality of the hospital where the patient was diagnosed is noted, including 3 migrant patients in fact infected in other countries but diagnosed in an Indian hospital (Table 2). When presupposing that the patients logically attended a hospital geographically close to the non-mentioned locality where they lived, the analysis of the altitude of the locality, an important aspect in fascioliasis transmission, shows very low altitudes of less than 500 m a.s.l. in a large majority of patients (35), whereas only 2 patients from the Kashmir Valley were infected at altitudes higher than 1000 m a.s.l.

One key question refers to the specific diagnosis. The majority of fascioliasis patients (31) were diagnosed as infected by *F. hepatica*, whereas only six were noted to be infected by *F. gigantica* and only one was molecularly studied and, interestingly, classified as an admixed *Fasciola* hybrid. Another great number of patients (17) remained without specific diagnosis (Table 2). Interestingly, infection by multiple flukes, i.e., more than three flukes, was only described in five patients. In one article, infection by *Fasciola* was also confused with *Fasciolopsis buski*.

The infection source was only noted in a few reports. Potential infection sources noted included eating fresh raw vegetables (in 6 cases), being related to cattle rearing (2 cases), eating salad of raw vegetables from the local market without washing or peeling (1), drinking water (1), bathing in a pond with cattle (1), and a rare case of liver transplant (1).

Regarding sex and age, the characteristics of the patients fit the expected results, with higher rates in females and a wide age range from early infections in children to old subjects, with the mean age peaking in adults (Table 2). The time elapsed between symptom onset and diagnosis is understandable when considering the insufficient awareness of physicians in India. The rare co-infection cases may be similarly explained.

The wide spectrum of different drugs used suggests that India was not ready to face patients infected by *Fasciola*. Once it was widely learned that triclabendazole was the drug of choice, health centers began to make efforts in obtaining this drug. The relative high number of patients in whom the current alternative drug nitazoxanide was used is evidently linked to the absence of triclabendazole availability in the country (Table 2).

**Figure 4:**
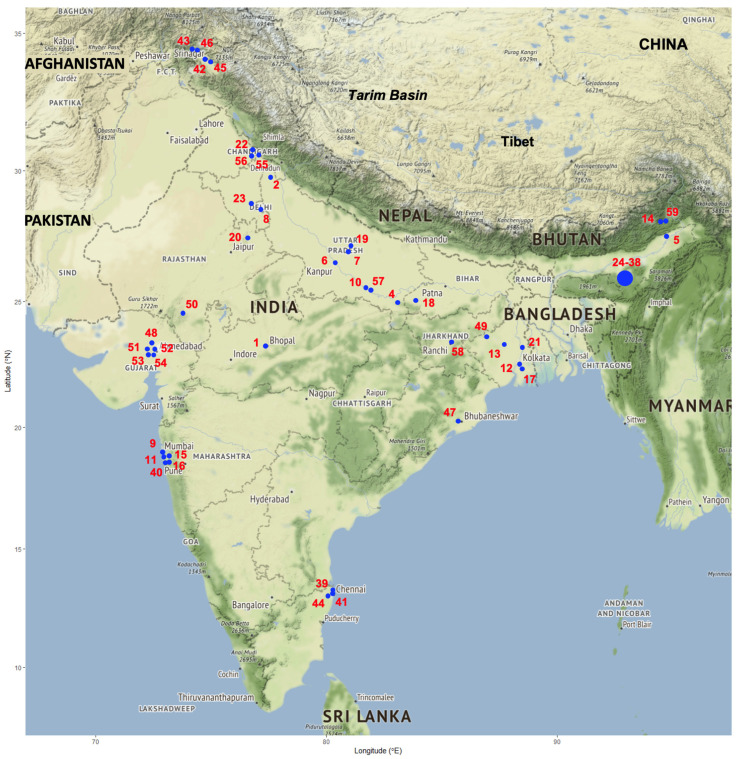
Map of India including state borders and neighboring countries, showing the localities of patients or the hospitals/health centers where they were diagnosed (in cases in which the original locality of the patient was not given in the published report). The numbers designating the 59 localities follow the chronological order of reports according to Table 1 (number 3 is not included because it concerns a traveler most probably infected in Egypt). Size of dot 24–38 is bigger because of the many patients tentatively located in Assam, representing northeastern India (the only detail given in the original publication). The localities were placed on the map using Google Earth Pro 7.3.6.9796 for Mas OS X. Original by S. Mas-Coma.

The analysis of the geographical distribution of the 59 aforementioned reports shows an *a priori* unexpectedly surprising distribution, which mainly concerns a fringe extending throughout all the northern part of the country from the West to the East, plus three main concentrations of reports in the western state of Gujarat and the big cities of Mumbai on the western coast and Chennai on the eastern coast (Figure 4).

This distribution includes reports from 18 states, with a high number of reports from northeastern India (Table 2). The three unpublished reports, notified to the WHO for the request of an Egaten^®^ donation for compassionate treatment of the respective cases, fit the aforementioned geographical framework: (i) a male patient diagnosed in Ahmedabad, Gujarat; (ii) another female patient who lived in Asansol, West Bengal; (iii) and a 5-year-old child patient diagnosed in Chandigarh, Punjab.

Concerning clinical aspects, several reports refer to patients suffering rare manifestations, such as a case presenting with cutaneous fascioliasis, and others with severe symptoms and complicated pictures. The following severe pictures have been reported: two cases of biliary colic, and single patients presenting with cirrhosis, paraplegia, vomiting and depression, severe paroxysmal epigastric periumbilical pain requiring hospitalization for two months, features of peritonitis and sepsis, vomiting associated with vertigo, death in a child surgically treated for a graft transplant, and a patient suffering from an advanced inoperable gallbladder carcinoma.

### 3.2. Climatic Analyses

The Spearman’s correlation found between the original precipitation data and the time series extracted from the continuous sources was significant in every case (*p*-value < 0.05) and presented the following correlation values: CHELSA, Spearman’s rho 0.11; GPCP, Spearman’s rho 0.90; CRU TS, Spearman’s rho 0.89. The precipitation data from CRU TS were selected for further analysis due to their finer spatial resolution and unit similarity compared with those from the GPCP database.

A total of ten meteorological stations were selected because of their appropriate geographical location regarding the geographical distribution of the fascioliasis cases (Figure 4), and they are illustrated in Figure 1 and Table 1: meteorological station No. 1 = Agartala; 2 = Ahmedabad; 3 = Amritsar; 4 = Balasore; 7 = Bhopal Bairagarh; 10 = Santacruz Mumbai; 20 = Jaipur Sanganer; 25 = Lucknow Amausi; 27 = Madras Minambakkam; 31 = Patiala.

**Figure 5:**
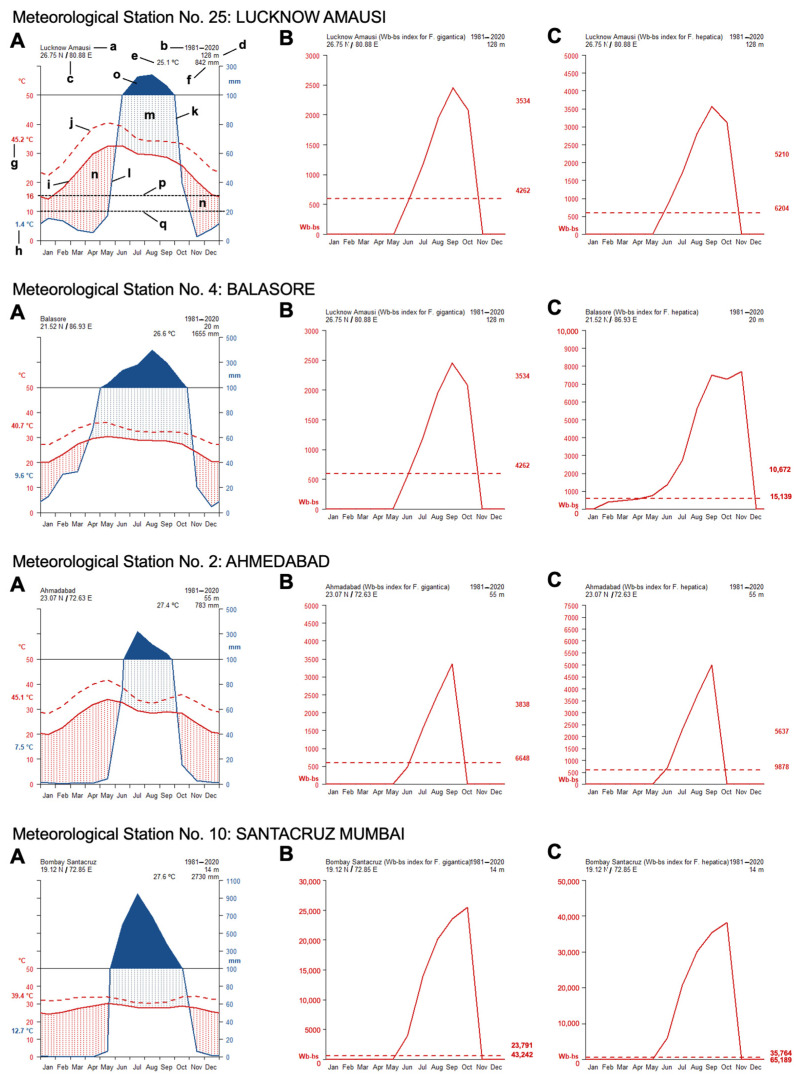
Analysis of the climate characteristics of the selected meteorological stations, representing four areas where many fascioliasis patients were diagnosed (see Figure 1 and Figure 4). (**A**) Climate diagrams showing plots including (a) the station; (b) the period of years represented by the data; (c) the geographical coordinates in decimal degrees; (d) the altitude in meters; (e) the mean yearly temperature; (f) the mean yearly precipitation; (g) the mean maximum temperature during the warmest month; (h) the mean minimum temperature during the coldest month; (i) the mean monthly temperature curve; (j) the mean maximum monthly temperature curve; (k) the potential evapotranspiration, as originally proposed [70]; (l) the mean monthly precipitation curve; (m) the wet season; (n) the dry season; (o) the months when the mean monthly potential evapotranspiration exceeds 100 mm; (p) the 16 °C temperature threshold, below which fascioliasis transmission due to *F. gigantica* is unlikely; (q) the 10 °C temperature threshold, below which fascioliasis transmission due to *F. hepatica* is unlikely. (**B**) Mean monthly values of the Wb-bs fascioliasis risk index for *F. gigantica* during the 1980–2020 period. (**C**) Mean monthly values of the Wb-bs fascioliasis risk index for *F. hepatica* during the 1980–2020 period. Original by P.F. Cuervo.

**Figure 6:**
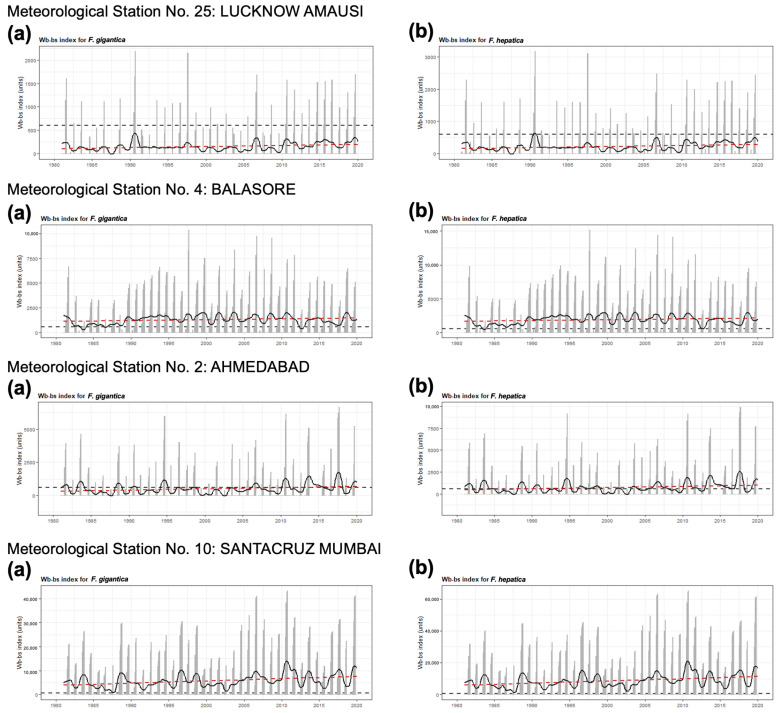
Gradually and progressively increasing values of the Wb-bs fascioliasis risk index during the 1980–2020 period in selected meteorological stations representing four areas where many fascioliasis patients were diagnosed (see Figure 1 and Figure 4). (**a**) Wb-bs risk index for *F. gigantica*. (**b**) Wb-bs risk index for *F. hepatica*. Original by P.F. Cuervo.

**Figure 7:**
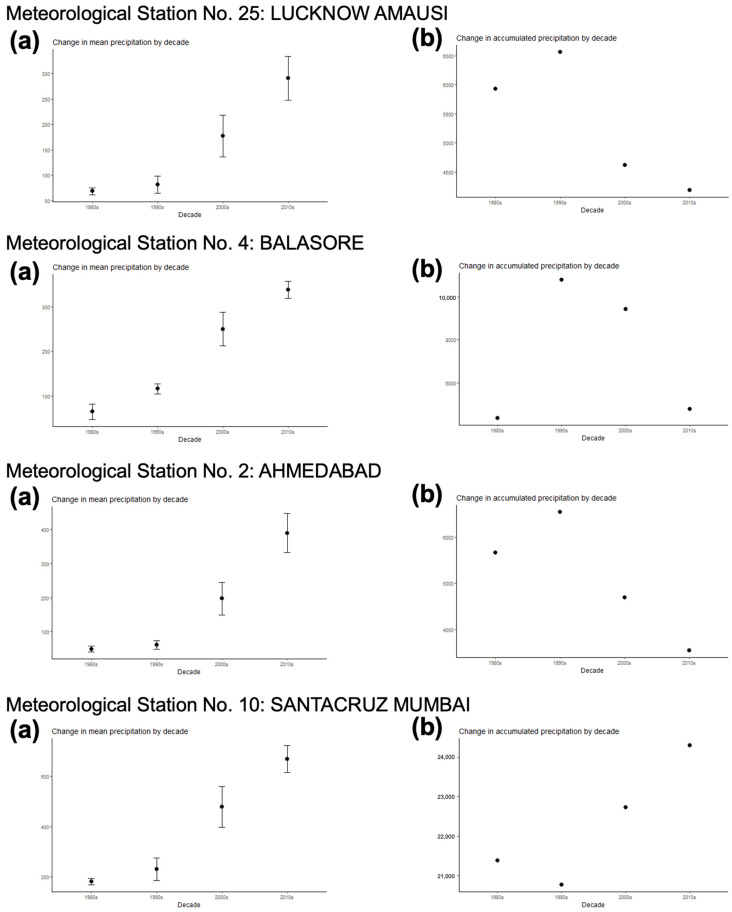
Analysis of precipitation in the selected meteorological stations during the period from 1980 to 2020, representing four areas where many fascioliasis patients were diagnosed (see Figure 1 and Figure 4). (**a**) Change in mean precipitation by decade. (**b**) Change in accumulated precipitation by decade. Original by P.F. Cuervo.

- The analytical studies have allowed us to understand the influences of climate factors on the transmission of fascioliasis throughout India and of climate change on the emergence of human fascioliasis in India:
- The climate diagrams covering the 1980–2020 period show that temperatures are higher than the minimum fascioliasis transmission thresholds of both *F. gigantica* and *F. hepatica* along a whole-year period (Figure 5A).
- The Wb-bs climatic forecast index analyzed throughout the aforementioned four-decade period indicates that fascioliasis transmission in India follows a marked seasonal pattern, including a potential transmission window from June–July up to October–November, with peaks between September and October. This applies for both *F. gigantica* (Figure 5B) and *F. hepatica* (Figure 5C).
- The analysis of the monthly values of the Wb-bs risk index along the 1980–2020 period shows a gradual, progressively increasing value for both *F. gigantica* (Figure 6a) and *F. hepatica* (Figure 6b) in the meteorological stations located in the four areas where most human fascioliasis patients have been diagnosed: meteorological station No. 25 of Lucknow Amausi (Uttar Pradesh), station No. 4 of Balasore (in Odisha, very close to West Bengal), station No. 2 of Ahmedabad (Gujarat), and station No. 10 of Santacruz Mumbai (Maharashtra).
- The aforementioned marked seasonality and progressively increasing Wb-bs value therefore appear to be related to precipitation and surface water availability, allowing for lymnaeid snail vector activity and liver fluke transmission, and, consequently, human fascioliasis infection risk. An evident pattern is observed when considering the mean monthly precipitation by decade (Figure 7). The mean monthly precipitation presents a marked increase in every meteorological station, and is particularly evident after 1980 (Figure 7a), whereas the total accumulated precipitation by decade evinces a negative trend in almost every location assessed (Figure 7b).

A few exceptions are exemplified by station No. 10 in Santacruz Mumbai, where the increased precipitation values in the last two decades may easily be ascribed to its proximity to the sea and probable increasing sea surface temperature due to the general global warming phenomenon, as seen in many other places in the world. The trend observed in the majority of the aforementioned selected meteorological stations indicates that precipitation is generally decreasing, but it also suggests that when it rains, the precipitation events are of greater magnitude. Consequently, the scarce temporal sources of water may persist for longer, concentrating the populations of both the lymnaeid snail vectors and the livestock reservoir hosts, thereby enhancing fascioliasis transmission.

### 3.3. Geo-Historical Assessments

In a country of such a large size and climatic pattern heterogeneity (Figure 1) as India, different types of livestock management are used depending on zones and areas according to their physiographic characteristics, topographical features, and climatic conditions, allowing for sufficient pastures (Table 3 and Table 4): (i) vertical altitudinal transhumance, (ii) horizontal plain transhumance, (iii) nomadic movements (long- or mid-distance herd movements, including both intra- and interstate displacements), (iv) local pastoralism (daily moving of ruminant herds around a central base camp where animals overnight), and (v) farming (e.g., animals stabled within fenced areas, including pasture fields).

Around 50 million people are involved with livestock management in India nowadays. Many different transhumant, nomadic, and pastoralist groups and farmers widely follow this type of lifestyle throughout India today (Table 3 and Table 4). Historical records indicate, moreover, that these ways of life have been maintained in India for thousands of years, since very ancient times. Many old ethnic groups are involved in human-guided movements of livestock across India (Table 4).

#### 3.3.1. The Grand Trunk Road

**Figure 8:**
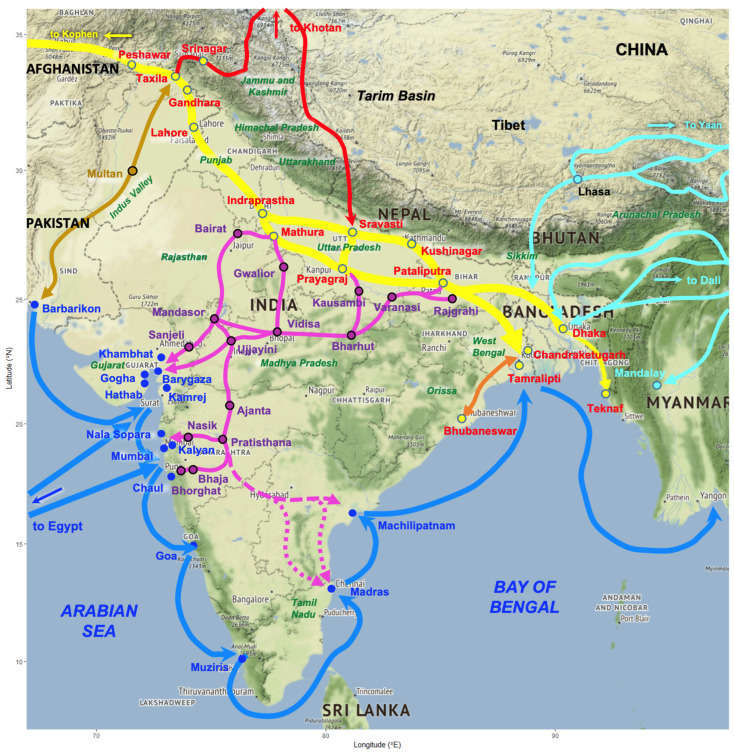
Map of India showing the past routes and main nodes (noted with the old names of the localities) used for human-guided movements of pack animals. Yellow: the Grand Trunk Road (formerly known as Uttarapatha) coming from Near East Asia through Afghanistan and Pakistan and eastward leading up to present West Bengal, Bangladesh, and the far seaports of Tamralipti and Teknof. Lilac: Daksinapatha route (originally from Varanasi and Kausambi southward up to Pratisthana) and its secondary routes, connecting (i) northward with the Grand Trunk Road nodes of Mathura, Prayagraj, and Pataliputra, (ii) westward with western seaports at the Arabian Sea, and (iii) southward with seaports at the Bay of Bengal during Mughal times (dashed lines). Blue: routes of the Maritime Silk Road. Dark blue: seaports of the Maritime Silk Road. Red: northward connections with the Silk Road. Light Blue: eastward connections with the Tea-Horse Road. Brown: western secondary connection between the seaport of Barbarikon and the node of Taxila of the Grand Trunk Road in Pakistan, and the eastern secondary connection between Bhubaneswar and the far ends of the Grand Trunk Road and the Tea-Horse Road through present-day Bangladesh. Data on this map were derived from many historical sources. Original by S. Mas-Coma.

Southern Asia was involved in these long-distance movements of pack animals since very early, by following the routes of the Grand Trunk Road (formerly known as Uttarapatha Road, or the northern route) (Figure 8) from the southern plains of Iran through Afghanistan and Pakistan, taking advantage of the Khyber Pass, a mountain pass between the Nangarhar Province of Afghanistan and the Khyber Pakhtunkhwa province of Pakistan.

**Figure 9:**
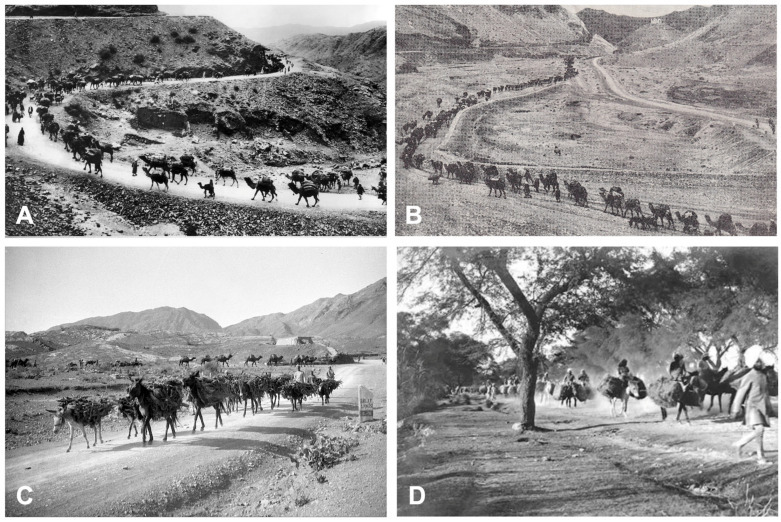
Long caravans of human-guided pack animals along the Grand Trunk Road illustrating the magnitude of animal transport involved. (**A**) Caravan composed by mainly camelids (dromedaries) and a few cattle (see bottom right) (ca 1925); (**B**) caravan including mainly dromedaries and a few donkeys (see bottom right) (ca 1930); (**C**) caravan of donkeys and mules in the forefront and a second caravan of dromedaries in the background; (**D**) caravan including numerous donkeys. (**A**,**B**): Old historical photographs; image credit: CSU Archive, Everett Collection Historical, Alamy Stock Photo, Abingdon, UK. (**C**) Old historical picture reproduced from a world trip by photographer James Dearden Holmes, 1925–1927, through Ninskaphotos, East Grinstead, UK. (**D**) Old historical picture available at the free online blog “The Grand Trunk Road”, posted by Alvin Plummer on 16 November 2020, in Stellar Reaches (A Fair Use Fanzine for Traveller), and designed with WordPress.com (<<https://stellarreaches.wordpress.com/2020/11/16/the-grand-trunk-road/>>–contents for non-commercial use only; accessed on 16 October 2024). Pannel composition by S. Mas-Coma.

These routes continued eastward throughout northern India and Bangladesh, where they linked with the Tea-Horse Road through Myanmar and China (Table 3, Figure 8). The magnitude achieved by the pack animal caravans following the long Grand Trunk Road is illustrated in Figure 9. The numerous and thousand-year-long movements that occurred from West to East and from East to West across these routes explain the pronounced genetic mixture found in the populations throughout this wide South Asian region.

#### 3.3.2. Southward Connections from the Grand Trunk Road

In India, extensive routes ran southward from several nodes of the Grand Trunk Road (Mathura, Prayagraj, Pataliputra) for thousands of years. The main route (formerly known as Daksinapatha Road, or the southern route) covered the northern nodes of Rajgrahi, Varanasi, Bharhut, Vidisa, and Ujjayini, subsequently southward to Ajanta and Pratisthana (now Paithan) (Table 3, Figure 8).

Two aspects should be highlighted. On one hand, this Daksinapatha Road had connections with many seaports of the Maritime Silk Road at the Arabian Sea, mainly around the Gulf of Kambay in Gujarat state, where the seaport of Barygaza was the most important, although other neighboring ports were also used throughout its history (Figure 8). More southern seaports at the Arabian Sea in the state of Maharashtra, among which that of Nala Sopara seems to have been the one most used by trade ships, were also running links with the southern far node of Prathistana of the Daksinapatha Road.

On the other hand, the Daksinapatha Road expanded farther southward to connect with eastern seaports of the Bay of Bengal, among which the routes down to Madras (present-day Chennai) began to develop during Mughal Empire times from the year 1526 (Table 3, Figure 8).

#### 3.3.3. Northward Connections from the Grand Trunk Road

The Grand Trunk Road connected with the northern mountainous Himalayan altitudes through: (i) the Chinese node of Khotan of the very long Silk Road in northern Asia, with nodes such as the Pakistani Taxila and the Indian Sravasti (Figure 8); (ii) many altitudinal transhumant activities in present-day western states such as Jammu, Kashmir, Himachal Pradesh, and Uttarakhand (Table 3); (iii) northward with China and also Nepal and Bhutan through Sikkim and the eastern branch of the Tea-Horse Road through the Tibetan Lhasa (Figure 8); and (iv) routes of vertical altitudinal seasonal transhumance in the eastern state of Arunachal Pradesh (Table 3, Figure 8).

#### 3.3.4. Geographically Extreme Connections from the Grand Trunk Road

In Pakistan, a route ran along the Indus valley between the node of Taxila of the Grand Trunk Road and the seaport of Barbarikon at the Arabian Sea (Figure 8).

In eastern India, the locality of Bhubaneswar in the state of the old Orissa (present-day Odisha), close to the seashore of the Bay of Bengal, established, from the 2nd century BC, mainland trade routes to foreign countries such as Myanmar, Thailand, and Vietnam, running through present-day Bangladesh (Figure 8), as well as maritime connections with Sri Lanka, Sumatra, and Bali.

## 4. Discussion

### 4.1. Human Infection Reports

Reporting articles refer to a low number of fasciolid infections and highlight the lack of baseline knowledge about human fascioliasis in India. This is understandable when considering the great dispersal of the reporting articles published in journals of reduced diffusion, such as journals of universities, local journals, journals of given hospitals, postgraduate journals, or books of zoological studies, together with the detection of cases according to a sporadic pattern in their geographical distribution, hospitals for case care, and different physicians involved in case diagnosis.

In India, human infection cases were very rare before the year 2005. The consequent logical general unawareness of physicians, health professionals, and diagnostic centers about human fascioliasis in this country sheds light on the usual briefness of the reports. The knowledge of human fascioliasis in India only relies on geographically isolated infected patients who are diagnosed and treated in different hospitals and health centers. No field survey looking for human infections has ever been performed in India. This is understandable when considering the so-far apparent lack of human infection risk areas, where such a survey could be justified.

There is a present emerging trend of animal fascioliasis and consequent increasing human infection risk throughout southern and southeastern Asia, from Pakistan in the West to Vietnam in the East. This trend in Bangladesh and northeastern India has been linked to: (i) ruminant importation from other countries because of the increasing demand of rapidly growing human populations; (ii) numerous livestock movements, including transborder corridors, due to uncontrolled small-scale household farming practices; and (i) the man-made introduction of *F. hepatica* with imported livestock into an area originally endemic for *F. gigantica*, leading to frequent hybridization [18]. The comparison of the epidemiological situations has demonstrated that this emergence scenario differs according to subregions. In Pakistan, it is linked to increasing monsoon rainfall due to climate change, combined with the impact of an extensive irrigation system [20]. Opposite to this, in Vietnam, a long-term multidisciplinary study has proven that climate change has played no influence on the countrywide spread of human fascioliasis, and that the spread of both liver flukes and lymnaeid vectors was indeed caused mainly by very numerous intranational and terrestrial trans-border human-guided movements of livestock, as well as overseas livestock importation from exporting countries of other continents [16].

The present review also highlights the insufficient information furnished by the reports, mainly concerning four crucial aspects:

(A) Specific diagnosis: In patients in whom the causal agent was classified at species level, the high number of cases ascribed to *F. hepatica* is surprising. Indeed, the original *Fasciola* species of the whole subcontinent of India is *F. gigantica*, except in the northern high-altitude areas, where *F. hepatica* is known to occur, such as in (i) the western Kashmir valley, (ii) the area of Sikkim located between Nepal, Bhutan, and Bangladesh, and (iii) the eastern state of Arunachal Pradesh [1].

When analyzing *F. hepatica*-diagnosed cases, in most patients neither the description of flukes recovered by ERCP or surgery, nor the description of eggs found in stool samples or duodenal or bile aspirates, furnish information to confirm the specific diagnosis [23,49]. In several of these cases, however, a specific misdiagnosis with *F. gigantica* can be concluded and, in other cases, easily envisaged. In several such reports, a photograph of the fluke illustrates a digenean parasite showing parallel lateral walls of the body, a feature suggesting misclassification with *F. gigantica* [26,29,40,44,46,51]. In cases diagnosed by egg finding, egg size was not measured, nor was a scale included in the photograph of the egg [42,50,51], making verification impossible based on the extreme values of length and width of the fasciolid eggs in human infections [4,102]. Egg size was only measured in a patient from Arunachal Pradesh in whom the infecting fluke was molecularly classified as belonging to an admixed fasciolid hybrid [18,35].

Furthermore, the absence of species of the *Galba*/*Fossaria* group of vectors specific to *F. hepatica* throughout the warm low altitudes of India [103,104] does not support the existence of transmission foci of *F. hepatica*.

(B) Geographical origin of the infection: The second problem found in almost all reports concerns the absence of the locality where the patient was living. In several reports, only the state of origin of the patient is given [35,39,50,53,56], and for 15 patients diagnosed in the hospital of Chennai between 2010 and 2016, it is only noted that they were from “northeastern India,” without any detail about even the state [45].

This lack impedes (i) the assessment of an accurate distribution of the disease and (ii) the appropriate analysis of its transmission foci and epidemiology. For instance, patients diagnosed in the hospital of Chandigarh, in Punjab, could have originated from or been infected in neighboring northern hilly areas where the transmission of *F. hepatica* may occur, which are opposite to the lowlands of the state of Punjab which are adequate for the transmission of *F. gigantica* [43,54]. A similar comment may be applied to patients diagnosed in a hospital in Assam but who could have been infected in high-altitude areas of the neighboring northern Arunachal Pradesh [27].

(C) Potential infection sources: Another piece of information usually lacking concerns data about the infection source, obtained during patient anamnesis. This makes it difficult to assess epidemiological interpretations regarding the infection sources of highest risk in India. There has been no way, except in a few cases, to assess whether certain traditional foods, attitudes, habits, behavior, housing, social aspects, or professional activities, such as those of livestock management practices or farmers working in field cultures of vegetables needing intense irrigation, may be linked to disease transmission and infection sources in India, as is known in other countries [10].
(D) Family/community infections: Except in a case of a Gujarati male patient whose wife was also diagnosed as being infected by *Fasciola* [54], in no other report were efforts made to verify whether other members of the family or community were also infected by liver flukes. In fascioliasis, subjects living close to others share the infection probability because of the use of the same traditional foods, attitudes, habits, behavior, housing, social aspects, and livestock management practices, mainly in rural areas but also in urban areas where metacercaria-carrying vegetables are sold in uncontrolled city markets or people share the same piped water supply at home [4,10,105].

### 4.2. Clinical Pictures, Symptomatology, and Pathology

According to the manifestations, two types of cases may be distinguished: (i) patients presenting with the clinical pictures usually or typically caused by fasciolid worms [8,106], and (ii) cases presenting with severe symptoms and complicated pictures.

It should be considered that the symptomatic picture does not differ between infections by *F. hepatica* and those by *F. gigantica* [8,107], although the chronological disorders caused in different serum biochemical parameters demonstrated that *F. gigantica* is more pathogenic despite its delayed development [7].

Aspects to be taken into account in defining the present situation of human fascioliasis in India include the following features:

(A) Absence of massive infections: In no fascioliasis patient infected in India were numerous liver flukes reported. This resembles the situation in Vietnam [16,107], but differs from the high intensities found in children of countries such as Bolivia [10,108,109,110], Peru [111,112], and Egypt [105]. The usual low intensity in Indian patients may indicate the absence of vegetables carrying high concentrations of attached metacercariae and the absence of reinfections leading to fluke accumulation because of the lack of premunition in human fascioliasis [113], with both absences suggesting low local transmission rates. This would indeed fit a situation of the first step of an emerging trend, as concluded when compared to the long period before 1992 in which only two cases were reported (Figure 1).
(B) Early diagnosis of most patients: In 28 patients, the time elapsed between symptom onset and diagnosis is noted. In only six cases was it of one year or longer (12 months: 1 case; 18 months: 1; 2 years: 1; 3 years: 2; 5 years: 1). In the remaining cases, this interval varied between 10 days and 7 months. This early diagnosis suggests that many patients were probably (i) in the invasive, migratory or acute phase, which explains the absence of eggs in stool, and duodenal and biliary aspirates, and justifies the cases diagnosed based on the clinical pictures, image methods, and/or serological techniques, or (ii) at the beginning of the biliary, obstructive, or chronic phase. This would underlie the still relatively small size of the flukes recovered from the patients, which could justify potential confusion regarding still-growing specimens of *F. gigantica* with *F. hepatica*. The usual early diagnosis of patients in India resembles the situation in Vietnam, where early diagnosis was facilitated by radio broadcasting [16] and explains the extreme rarity of disorders that typically appear in the long-term advanced chronicity of fascioliasis [107]. The opposite situation of long-term delayed diagnosis was frequent in Argentina and explained extreme clinical pictures and surgical interventions due to lithiasis suspicion [114]. In non-endemic countries or areas, an early correct diagnosis avoids frequent misdiagnoses and unnecessary surgery [115].
(C) Higher infection rate in females: The analysis shows an infection rate 1.57 times higher in females (n = 33) than in males (n = 21). A higher prevalence rate in females is well known in human fascioliasis-endemic areas, such as in Vietnam [16], southern China [116], Egypt [117], and Peru [112]. Similarly, infection intensities estimated by fecal egg counts (eggs per gram of stool = epg) also proved to be higher in females in Bolivia [110] and Egypt [105]. Unfortunately, egg quantification was conducted in no Indian patients. Indian health centers will need to use a quantitative analysis tool such as the Kato–Katz test (or any other quantitative diagnostic technique) to quantify epg in the patients, mainly to assess the adequate treatment drug dose to avoid the potential risk of bile duct obstruction due to the accumulation of dragged flukes and consequent colic. A quantity of 300 epg was established by the WHO as the colic risk limit, so a divided drug dose should be administered when surpassed [4]. This limit was increased to 400 epg for the human hyperendemic area of the Northern Bolivian Altiplano, where massive infections were frequently detected in children [118,119]. Interestingly, however, biliary colic was reported in two Indian patients, a 40-year-old female from Saharanpur, Uttar Pradesh [24], and a 40-year-old male from Alwar, Rajasthan [41], in both of whom only one adult fluke was found after surgery and ERCP, respectively.
(D) Patient age peaking in adults: The age of infected patients in India showed a mean of 33.1 years. This agrees with the situation in Vietnam, where infection rates peak in the age groups of 31–40-year-old and 41–50-year-old subjects [16], and southern China, where the age mean is 38.54 ± 15.68 years [116]. Such age-related infection rates pronouncedly differ from human hyperendemic areas in Andean countries [110,112] and the Nile Delta in Egypt [117], where the highest infection rates concentrate in 5–15-year-old schoolchildren.

In addition, the diagnosis in a preschool child of only 30 months merits a comment because of the rare early infection [120]. This child, diagnosed in the Gandhi Medical College of Bhopal, was the third sibling of a vegetarian family living in the neighboring locality of Agarwal. This child, the only patient from Madhya Pradesh, was totally breastfed till the age of 21 months, and presented with fibrotic granulomas, referred to as childhood cirrhosis. Another sibling had already died due to “some liver disease” [23]. The reporting authors suggested that the infection source could have been the drinking of well water that was not boiled. Whether infected by vegetables or by contaminated water, all evidence suggests that human infection might have been underestimated in the child’s family and the community.

Several patients presenting with rare manifestations, severe symptoms, or complicated pictures also need specific analyses to clarify or highlight important aspects.

A 27-year-old female patient living in Lucknow, Uttar Pradesh, presented with symptoms indicating liver infection and a cutaneous picture of a serpentine red-colored vesicular lesion, from which a fluke was extracted after incision adjacent to the lesion [40]. Although originally reported as belonging to *F. hepatica*, the published photograph shows a typical elongate fasciolid worm with parallel lateral body walls, indicating that the parasite did in fact belong to *F. gigantica*. Ectopic fasciolid disorders are known in infections by both *F. hepatica* and *F. gigantica* [8], and cutaneous manifestations caused by *F. gigantica* have been described in Vietnam [121].

The case of a 34-year-old male patient diagnosed in a Rohtak hospital in Haryana is of interest because of the laparotomy intervention due to presenting with blunt abdomen trauma with features of peritonitis and sepsis [44]. Peritonitis is rare in fascioliasis and has only been reported in three other cases, namely in France [122], Turkey [123], and Vietnam [120].

In three Indian patients, manifestations were reported that are known in infections presenting with neurological, meningeal, and/or neuropsychic disorders [8]. Such pictures are caused by the proinflammatory peptide bradykinin, leading to blood-brain barrier leakages generated by alterations in the fibrinolytic and contact systems induced by plasminogen-binding proteins excreted/secreted by migrating juveniles and liver adult flukes of both *F. hepatica* and *F. gigantica* [124,125]. In a 30-year-old female patient diagnosed in a Lucknow health center in Uttar Pradesh, manifestations such as bilateral lower-extremity weakness and paraplegia were described [29]. In two patients diagnosed in a Mumbai center in Maharashtra, depression was reported in a 16-year-old female [33] and vertigo in a 54-year-old female [46], both manifestations underlying recurrent vomiting.

Two reports concerned exceptional cases. An article reported the death of a 6-year-old girl living on a farm rearing sheep and cattle in Morocco and who was surgically treated in a Chennai hospital in Tamil Nadu for a liver transplantation with an allograft from a Moroccan donor [47]. It should be considered that Morocco is a country with endemic areas of only *F. hepatica* [1] (regarding misdiagnosed F. gigantica in Morocco, see [126]). Returning to Morocco after effective transplantation and treatment, the patient suffered multiple episodes of cholangitis, with progressive graft dysfunction throughout the subsequent year. The child expired one month later in Morocco due to severe cholangitis and septic shock. This is the first reported case of fascioliasis transmitted through a donor graft, which was confirmed when verifying the infection by *F. hepatica* both etiologically by egg finding in stools and serologically by the positivity of an ELISA test [47]. As highlighted, transplant recipients are at higher risk of infections, are prone to having post-operatory disseminated diseases, may present with subsequent atypical manifestations, and may not show an adequate response to the treatment.

Finally, a 45-year-old female patient living in the rural area of Bhubaneswar and diagnosed in Chandrasekharpur, Odisha, was reported to be infected by four flukes recovered by ERCP and classified as *F. hepatica* [51]. However, the apparent parallel lateral body walls in the ERCP image suggest flukes probably belonging to *F. gigantica* (nothing can be concluded from the extracted pronouncedly contracted worms seen in another photograph and the absence of eggs in the stools). This, together with a repeated misspelling as *Fasciolopsis* in the article, suggests questionable aspects [51]. The interest of this report relies on the pathological observation of the advanced inoperable gall bladder carcinoma in this patient, presumably caused by the fasciolid infection, which does not fit the general belief about the lack of cancerogenic capacity of fasciolid trematodes compared to other hepatic trematodes of the genera *Opisthorchis* and *Clonorchis* [127].

### 4.3. Problems, Exceptions, and Clarifications

Several reports need specific clarifications, which should be taken into account for the correct assessment of human fascioliasis in India.

There have been fascioliasis cases diagnosed in India that indeed concerned patients infected in other countries. For instance, there is a case of a USA female traveler reported in 1993 [25] who presented with a coinfection by *Giardia intestinalis* and who spent the 1957–1975 period in Pakistan, the years 1981 and 1982 in India, and 1989 to 1991 in Egypt. The first symptoms appeared when in Cairo, Egypt, a country where this trematodiasis is a well-known public health problem caused by both *F. hepatica* and *F. gigantica*, including high prevalences [117] and infection intensities [105]. The analysis of the chronology of symptom appearance and diagnosis clearly indicates that this patient was infected in Egypt and not in India.

Another two reports in fact concerned the same 9-year-old male patient coinfected by *F. hepatica* and *Hymenolepis nana* and diagnosed in a hospital in Mumbai [36,37]. This child was an immigrant from Kolchibahur, Nepal, a country where both *F. hepatica* and *F. gigantica* are also present [1] and have been diagnosed in human infections [128].

Other such cases concerned a 37-year-old vegetarian male and his 27-year-old wife living in Gujarat state and diagnosed in a hospital in Ahmedabad, after lived for four years in Kampala, Uganda [53]. Although fasciolid eggs were found in the duodenal aspirate of the male and ascribed to *F. hepatica* (the wife was only clinically diagnosed), this specific diagnosis cannot be supported due to the absence of egg measurements and the absence of scale in the figure. Moreover, the fasciolid species known to be present in Uganda, namely *F. nyanzae* in hippopotamuses and *F. gigantica* in domestic ruminants [1], do not suggest a potential infection by *F. hepatica* in Uganda. In this country, only on Mount Elgon can there be found a *Galba*/*Fossaria* species, *L. mweruensis*, able to transmit *F. hepatica*, although *F. hepatica* was not found in that area [129]. This suggests that the report of *F. hepatica* infection in a few cattle slaughtered in the Kampala City abattoir was either misdiagnosed or imported from outside, such as from the neighboring Kenya highlands or other countries [130]. Indeed, such importations in southeastern African countries are well known to occur nowadays [1].

The two cases of an *F. hepatica*-infected 32-year-old male and an *F. gigantica*-infected 20-year-old female from the Kashmir Valley were both repeatedly reported in two different articles [48,50]. Another article included the report of 16 fascioliasis cases diagnosed in a hospital in Chennai between 2010 and 2016, among whom only one case was local, whereas the remaining 15 patients were from northeastern India, without detailed information about their geographical origin [45].

An interesting case of a 55-year-old woman from Arunachal Pradesh was reported in two articles. The first article, published in 2012, concerned the report and description of this case [35]. The second one, published in 2024, molecularly proved the fasciolid eggs collected from this same patient to belong to an admixed fasciolid hybrid [18].

Lastly, the report of fascioliasis in a 20-year-old pregnant woman who presented at Ballabhgarh, India, in labor, with gastrointestinal symptoms of 3 days’ duration, should be highlighted. This patient, treated for emergency lower-segment caesarean section, was indeed infected by *Fasciolopsis buski* (and not *Fasciola*, as noted in the title of the article), as verified after vomiting a specimen of this intestinal trematode species, which explains why praziquantel was effective [131].

### 4.4. Assessments by Combining Climatic and Geo-Historical Analyses

The comparison of the results of the case review, climatic analyses, and geo-historical analyses allows us to clarify (i) the fluke agent causing the human infections, (ii) the risk seasons for human infection, and (iii) the geographical zones and areas of high human infection risk.

#### 4.4.1. Species of *Fasciola* Infecting Humans

The fasciolids infecting humans in India pose the question of their specific classification. The majority of case reports were noted to concern *F. hepatica*. This posed no problem in the past because *F. gigantica* was thought to only rarely infect humans. However, present knowledge has recently proven that *F. gigantica* may also cause significant public health problems. The countrywide spread of human fascioliasis caused by *F. gigantica* in Vietnam [16], and the clinical picture it causes in humans [107], have recently changed the conception.

Our climatic analyses demonstrate that almost the entire territory of India is susceptible to the transmission of both fasciolid species, excepting very high altitudes of the Himalayan chain, where the cold temperatures pose a barrier against *F. gigantica*. Moreover, the absence of *Galba*/*Fossaria* lymnaeids explains why *F. hepatica* has had difficulty being transmitted throughout the wide lowlands of India [103,104], excepting:

- The northwestern altitude areas of the Kashmir Valley, Himachal Pradesh, and its foothills, where the past connections through the Silk Road facilitated the arrival of this fasciolid species and its lymnaeid vectors, essentially *Galba truncatula* [1,83,132,133].
- The northeastern altitudes of Arunachal Pradesh, where the past connections through the Tea-Horse Road also allowed for the arrival of *F. hepatica* and lymnaeid-transmitting species today known in Nepal, southern China, and southeastern Asia [1].
- Northeastern Indian lowlands and low altitudes surrounding Bangladesh, mainly West Bengal, Sikkim, Assam, and Arunachal Pradesh, where livestock importation has been seen to underlie the introduction of *F. hepatica* from foreign countries of other continents that are endemic only for *F. hepatica* [17]. This man-made activity leads to fluke hybridization, giving rise to nuclear rDNA admixed *Fasciola* hybrids infecting both domestic ruminants [17] and humans [18] in the aforementioned states of India. In agreement with this scenario, intermediate forms have also been found in high-altitude areas of Sikkim [134]. The consequent increase in fascioliasis transmission rates is most probably related to the high number of liver fluke-infected patients reported from this northeastern part of India [45].

It should moreover be considered that altitudinal seasonal transhumance, horizontal lowland seasonal transhumance, and nomadic pastoralism are practiced in many states of India (Table 3 and Table 4). These human-guided livestock movements imply that many persons involved in such movements are (i) at high risk of fascioliasis infection because of their closeness to the ruminant reservoirs and (ii) may be infected in one place but diagnosed as having been infected by the liver fluke in another place. Indeed, most, if not all, of these seasonal movements used to follow identical transects that are populated by lymnaeid snail vectors, after spreading and colonization by these snails occurred during past movements [1].

Among the many lymnaeid species and forms described throughout India [103,104], the transmission of the warm lowland-preferring fasciolid species *F. gigantica* appears to be linked to two radicine species widely distributed throughout the lowlands of the country:

- *Radix luteola* is a mid-sized lymnaeid (adults of around 1.5–2 cm in length) recently proposed to be included in the genus *Racesina* [135]. The life cycle of this radicine species fits environmental conditions of pH 7 and a temperature between 20 and 35 °C, and is unable to complete its life cycle at 10 °C and 15 °C [136]. The preference for warmer temperatures agrees with the hot conditions reached by the lowlands throughout India up to the lowest latitudes [137,138]. This species shows an amphibious trend, which underlies its preference for irrigated lands and channels, such as, for instance, in rice fields, although it may also be found in ponds [138]. *Radix luteola* is often found in temporary water bodies that dry up in summer, surviving unfavorable conditions by burying inside the mud. When compared to lymnaeid species that are well known to be involved in human infection, such as the amphibious species *G. truncatula* in Europe, *Lymnaea neotropica* in South America [139], and *Radix viridis* in Vietnam [16], the similar behavioral characteristics of *R. luteola* suggests that it is the most important vector regarding human infection in India.
- The superspecies *Radix auricularia* includes species or varieties differing in shell forms, among which the Indian *R. acuminata* has recently been proposed to be included in the South Asian species *Radix rufescens* [135]. *Radix acuminata* is a more aquatic and large-sized lymnaeid species (with adults reaching a length greater than 2 cm), typically occurring in permanent water bodies with abundant vegetation, such as in small to large ponds, where it reaches very high prevalences of infection by *F. gigantica* in ponds frequented by buffaloes [140]. The life cycle of this radicine species shows a longer survival and higher multiplication rates at the lower temperatures of 15 °C and 20 °C, whereas its lifespan and egg production are markedly reduced at the higher temperatures of 25 °C and 30 °C [141]. This species, and also *R. luteola*, have been reported in the southernmost areas of India, such as Tamil Nadu [142]. The ecology of *R. acuminata* favors human infection by freshwater drinking and is suggested to be less involved in human infection.

#### 4.4.2. Transmission Seasonality and Evolution of the Yearly Infection Risk

Our climatic analyses show that a vast territory of India is *a priori* suitable for the transmission of both *Fasciola* species. Although this fits the present knowledge about fascioliasis in livestock throughout Indian states (Table 4) [143], the distribution of human fascioliasis shows an evident restriction to only given zones (Figure 4).

Our analyses demonstrate that (i) fascioliasis transmission in India follows a marked seasonality from June–July up to October–November, with peaks in September–October, coinciding with the summer monsoon season, and that (ii) the Wb-bs index shows a gradual, progressively increasing value for both *F. gigantica* and *F. hepatica.* This marked seasonality and the progressively increasing value of fascioliasis transmission risk are related to:

- Higher temperatures favoring the warm condition-preferring species *R. luteola*, which is the lymnaeid species showing ecological and behavioral characteristics most appropriate for fascioliasis transmission.
- A marked increase in the mean monthly precipitation, which has been evident since 1980, which is due to fewer rainy days but more days of extreme rainfall events, leading to increasing amounts of rain per event and, consequently, more frequent floodings and increased surface water availability. This favors population dynamics of amphibious lymnaeids such as *R. luteola* and fascioliasis transmission in temporary transmission foci. Despite the generally decreasing precipitation, scarcer temporal sources of water may persist for longer, concentrating the populations of both the lymnaeid snail vectors and of the livestock reservoir hosts and thereby favoring fascioliasis transmission. Public health problems posed by human fascioliasis have already been reported from semiarid–arid areas, where water availability depends on located water sources. In such drought situations, disease transmission factors are concentrated in small areas where humans and animals go for water supply, vegetable cultures, and livestock farming [144], epidemiologically resembling desert oases [145].

Wide climatological analyses catalogue India as one of the most vulnerable countries in the world regarding climate change [146], including water stress, heat waves and drought, severe storms and flooding, and the associated negative consequences on health and livelihoods.

Our climate analyses concerning areas where fascioliasis patients were diagnosed fully agree with the trends observed throughout the whole of India [147]. All models show a trend of general warming of mean annual temperature and decreased range of diurnal temperature. India’s average temperature rose by around 0.7 °C during 1901–2018. In 1986–2015, the temperatures of the warmest day and the coldest night of the year rose by about 0.63 °C and 0.40 °C, respectively. Both the frequency and the average duration of heat waves have pronouncedly increased in the April–June period throughout India [147].

**Figure 10:**
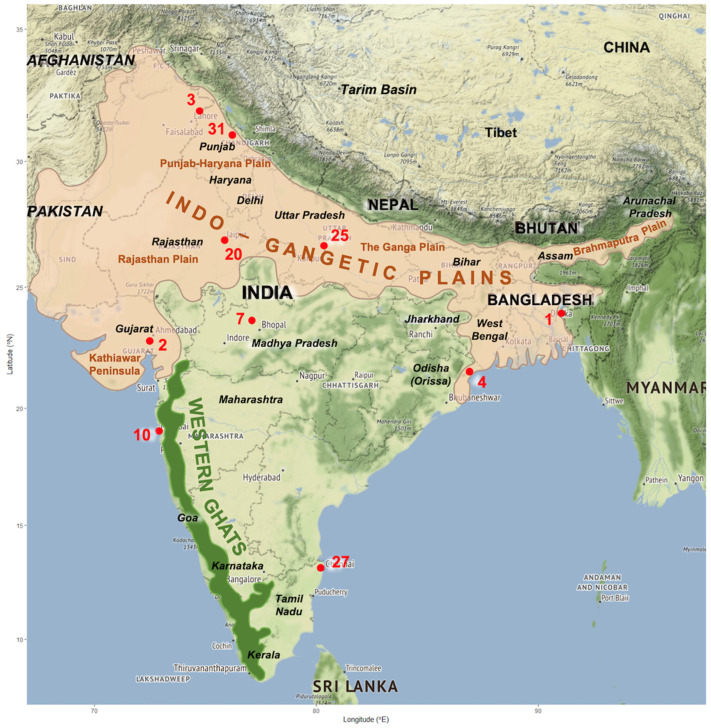
Map of India showing the extent of the lowlands included in the so-called Indo-Gangetic Plains, covering the Brahmaputra Plain, the Ganga Plain, the Punjab-Haryana Plain, the Rajasthan Plain, and its southeasternmost extension of the Kathiawar Peninsula in the state of Gujarat (in pink), which includes the vast majority of the localities where the fascioliasis patients lived and/or where diagnosed; the western mountain chain range of the Western Ghats (in dark green), whose northern range was crossed by the routes of the Daksinapatha Road for the connections with the western seaports used in the Maritime Silk Road; and the ten meteorological stations (in red) selected in consideration of the geographical distribution of the human fascioliasis cases reported and from whose data the forecast indices were calculated for each month of each year. Original by S. Mas-Coma.

Models also show enhanced precipitation in India. This increased precipitation, including monsoonal rains, is expressed as fewer rainy days but more days of extreme rainfall events, with increasing amounts of rain in each event, leading to significant flooding. The summer monsoon precipitation (June to September) in India has declined by around 6%, with notable decreases over the Indo-Gangetic Plains and the Western Ghats (Figure 10), including a shift toward more frequent dry periods and more intense wet periods during the summer monsoon season. Nomadic pastoralism is gradually adapting to the environmental modifications induced by this climate change [148].

The Indo-Gangetic Plains cover the extensive north–central region of the Indian subcontinent, stretching from the easternmost Brahmaputra river basin along Arunachal Pradesh and Assam and the Ganges river in West Bengal and Bangladesh in the East, to the Punjab-Haryana plain and the Indus River valley and southward to the Rajasthan Plain and Gujarat state in the West. All in all, this wide region contains the most densely populated areas of India (Figure 10). The Western Ghats is the mountain chain that stretches 1600 km and runs parallel to India’s western coast, approximately 30–50 km inland and with altitudes of up to 2695 m, traversing the states of Gujarat, Maharashtra, and southward to Kerala and Tamil Nadu (Figure 10).

Interestingly, 51 localities where the patients were diagnosed out of the total of 55 cases (92.7%) are in the region of the Indo-Gangetic Plains and the northern Western Ghats (compare Figure 4 and Figure 10). Only the case from Agarwal, diagnosed in Bhopal [23], and three cases diagnosed in Chennai [45,47,49], fall outside this geographical frame.

The cases diagnosed in Mumbai, discounting the immigrant from Kolchibahur, Nepal [36,37], only concern one patient, with a history of having traveled to Rajasthan a few months prior [46], plus only two other cases [31,33]. This means that the large majority of cases are connected to the Indo-Gangetic Plains. Mumbai is located on the western coast, and therefore the topography and proximity to the sea and the northern Western Ghats surrounding the Mumbai area determine the temperature of the zone [149]. Seasonally, spatial patterns of sea surface temperature indicate a large northward spread of temperatures greater than 28 °C from winter to summer in the Arabian Sea [150].

Regarding the wide remaining part of India, the overall decrease in seasonal summer monsoon rainfall during the last few decades has led to an increased propensity for droughts. Both the frequency and the spatial extent of droughts have increased in areas over central India, the southwest coast, and the southern peninsula [147].

#### 4.4.3. Geographical Distribution of Human Infection Hotspots

A question is posed when considering that animal fascioliasis is distributed throughout all Indian states (Table 4) and that the climatic conditions are suitable for fascioliasis transmission throughout the country, whereas human fascioliasis shows a restricted geographical distribution (Figure 4). Which factors underlie this difference between animal and human fascioliasis?

The fascioliasis distributional frame delineated by the old roads throughout the Asian continent offers a baseline for understanding this difference. Human-guided movements of pack animals along very long distances and for thousands of years have been demonstrated to underlay the spread of *F. hepatica*, *F. gigantica*, and lymnaeid snail vectors, and are known to correlate with present areas of high human fascioliasis infection risk [1].

The Neolithic period of 12,000–11,000 BC led to a revolution in the way of life of humans, mainly in the so-called Fertile Crescent, in the Near East region of Asia. The domestication of animals and the cultivation of nutritional vegetables gradually divided the traditional hunters into two types of human groupings: (i) sedentary trends of stable human populations depending on local farming practices of livestock in areas where herbivore ruminants found grazing sources from available pastures along the whole year, and (ii) moving human herder groupings guiding ruminants either vertically throughout different altitudes, allowing animals to graze in lowland plains during the winter cold season and in mountainous highlands during the warm summer season, or horizontally along more or less long lowland trajectories, leading animals from areas where pastures become insufficient due to dryness to other distant areas where pastures are still available due to sufficient rainfall patterns. These moving ways of life underlie transhumant pastoralism and nomadic pastoralism, respectively [151].

Such analyses about past routes repeatedly followed by human-guided movements of pack animals for hundreds or thousands of years are useful to understanding the current distribution of fascioliasis and liver fluke infection risk. Several studies have already demonstrated this usefulness, such as in: Uruguay, Argentina, and Chile [139,152] in South America; in Algeria in Africa [1,126]; and in Iran [115,153], Bangladesh [17], and Vietnam [16] in Asia.

The northern Silk Road, the southern Grand Trunk Road, and the Tea-Horse Road, as well as goods transport from the seaports of Arabian Sea and Bay of Bengal along the so-called Maritime Silk Road to their respective hinterlands and transect connections with the aforementioned big roads, are illustrative examples on the Asian continent [1].

All these old roads converged in India (Table 3, Figure 8). These past roads did not follow narrow ways or routes when running along lowland plains without topographic obstacles (such as mountain passes or gorges, river crossings, or known bridging points) or human-helping constructions for migrants (way stations, caravanserais, forts, small towns, shrine complexes) [154]. It is therefore better to speak about corridors whose lateral boundaries evolved over time. Moreover, for reports lacking the patient’s locality, it should be considered that the hospital location is useful, because patients usually look for health care in the proximity of their living places.

After taking into account the aforementioned two aspects, when comparing the distribution of fascioliasis patients (Figure 4) with the map showing the old roads throughout India and their exchange connections with neighboring countries (Table 3, Figure 8), the overlap appears evident. The lowlands of the Indo-Gangetic Plains include most of the geographical distribution of the patients. This indicates that the Grand Trunk Road and its southward connections were not only involved in the introduction of *F. gigantica* from the western Fertile Crescent and its subsequent spread in the past [1], but also assured long-term fascioliasis transmission by progressively linking lymnaeid populations to local freshwater environments repeatedly visited by ruminant reservoirs.

Outside of the Indo-Gangetic Plains, the case from Agarwal, diagnosed in a Bhopal health center (Figure 10), appears to be geographically linked to the Daksinapatha Road through a southward route connecting with the Grand Trunk Road for goods transport (Table 3, Figure 8). This was archeologically followed based on the discovery of amphorae in towns located along the transect [75].

The several cases in Gujarat also appear to be related to fascioliasis risk established long ago due to the interconnections between ship transport by the Maritime Silk Road using many seaports at the Gulf of Kambay and the Daksinapatha Road connecting with the Grand Trunk Road (Table 3, Figure 8). Fascioliasis prevalences found in livestock in Gujarat suggest the link of this disease with local transmission foci [155,156].

The two fascioliasis patient groupings in the western seaport of Mumbai (previously known as Bombay) at the Arabian Sea and in the eastern seaport of Chennai (formerly Madras) at the Gulf of Bengal are exceptions outside of the Indo-Gangetic Plains. Both these seaports and their close neighboring small seaports played important roles in the past of the Maritime Silk Road (Table 3, Figure 8). Several very old small seaports around the former Bombay were connected to the southward arm of the Daksinapatha Road by crossing the northern hills of the Western Ghats (Figure 8 and Figure 10) [75]. In its turn, the seaport of the former Madras was the end node of a tertiary–quaternary route southward, derived from the Daksinapatha Road and launched only quite recently during the Mughal Empire from the year 1526 (Table 3, Figure 8). This gave rise to connections with the Madras hinterlands by using pack animals for round-trip transportation of persons and goods arriving on the ships, and the subsequent spread of *F. gigantica* in livestock [157,158] and lymnaeid vectors in transmission foci [137,138,142] throughout Tamil Nadu.

The results of many studies using DNA sequencing and other molecular techniques of *Fasciola* in India do all agree with the overall distribution of *F. gigantica* throughout the lowlands of this country [134,159,160,161,162,163,164,165,166], as delineated by the past main Grand Trunk Road, the past secondary Daksinapatha Road, and the tertiary and quaternary routes (Table 3, Figure 8), according to the aforementioned archeological evidence and historical knowledge [1].

In northwestern India, where past connections with the northern Silk Road occurred (Table 3, Figure 8) [1], several studies on domestic ruminants in northern high-altitude areas are the only reports of *F. hepatica*, as in Kashmir Valley [167,168,169,170] and the state of Himachal Pradesh [83]. In northeastern India, where past connections with Nepal and China occurred along the Tea-Horse Road (Table 3, Figure 8), and thereby also with the northern Silk Road [1], intermediate forms have been reported in livestock in Sikkim [134], and molecularly verified hybrids have been identified as having infected a human in Arunachal Pradesh [18]. These studies have moreover highlighted the very recent introduction of *F. hepatica* with livestock imported through seaports of the Bay of Bengal from foreign exporting countries endemic for this fasciolid species and the uncontrolled livestock exchange between India and Bangladesh [17].

In other parts of India, climatic studies agree with the low prevalence in livestock (Table 4) and allow for the explanation of the absence or very low risk of human infection, despite the numerous transhumant, nomadic, and pastoralist groups guiding the movements of livestock reservoir species throughout many Indian states in the past and still nowadays [171,172], and which were surely involved in the spread of *Fasciola* and its lymnaeid vectors throughout the rest of India.

## 5. Conclusions

The combined consideration of the results obtained in the case review, climate analyses, and geo-archeo-historical assessments allows us to reach crucial conclusions that should be henceforth considered in India:

(A) Physicians in charge of fascioliasis patients should henceforth consider the following key points:
  - Adequate efforts should be made to reach a specific diagnosis by describing the morphology and length/width of adult worms if obtained by ERCP or surgery, and/or by describing and measuring the length/width of eggs if found in stools or bile/duodenum aspirates; moreover, authors of case reports are requested to add a scale in photographs illustrating worms and/or eggs.
  - When eggs are found in the patient’s stool, epgs should be quantified by using Kato–Katz or any other quantitative technique, and thereby assessing the appropriate drug dose to avoid post-treatment colic.
  - Physicians should consider a specific serological test, several of which are commercially available [4], for patients not shedding eggs; overlooked or misdiagnosed human infection cases in India cannot be ruled out.
  - The locality where the patient is living should be provided to obtain accurate information about the geographical risk of human infection.
  - In anamnesis, patients should be questioned about potential infection sources, i.e., which suspicious vegetables form part of their diet and the origin of these vegetables (sylvatic, agricultural fields, uncontrolled city markets), and water-drinking habits (from natural collections shared with livestock, wells, irrigation canals, streams or rivers, traditional beverages).
  - The patient should also be asked about other members of the family or community sharing food and drinking traditions [4], and carry out their diagnosis if the information suggests their possible infection.
  - The recently described complete clinical picture caused by *F. gigantica* [107] may be henceforth useful for clinicians in India.
(B) Epidemiologists should consider the conclusions of the present multidisciplinary assessments:
  - Current climate trends throughout India suggest a future increasing emergence of human fascioliasis.
  - The seasonal risk of human infection ranging from June–July up to October–November in India allows for the definition of prevention and control measures.
  - The geographical zones of high risk of human fascioliasis defined by archeological–historical analyses, mainly linked to the Indo-Gangetic Plains and corridors used by the old Grand Trunk Road and old Daksinapatha Road, but also in northern mountainous areas, as well as the hinterlands of western and eastern seaport cities involved in the past Maritime Silk Road [1], also allow for the design of specific control measures.
(C) Responsible government officers should also consider several conclusions for the necessary improvement in certain crucial items:
  - Meteorological stations should assure the continuity of climate factor measurement and make efforts to provide available data; climate change trends in India indicate a future worse situation, and meteorological data will be increasingly needed.
  - Measures should be implemented to reduce uncontrolled livestock exchange with neighboring countries and apply appropriate animal quarantines and treatments for livestock imported from foreign endemic countries to avoid *F. hepatica* introduction, risky fasciolid hybridization, or the introduction of lymnaeid vectors attached to the hooves of imported ruminants [16,17,18].
  - Human health administration officers should seek the official registration of triclabendazole for human use (Egaten^®^ from Novartis Pharma, Basel, Switzerland), and recommend drugs different from triclabendazole for animal treatment to avoid the appearance of resistance to this drug.

## Figures and Tables

**Table 1:** Meteorological stations analyzed in India for the period 1980–2020, and respective monthly data available. See the map in Figure 1 for the location of each meteorological station.

Location on Map	Station	International Station Code	Geographical Coordinates	Altitude (m)	Available Data (%)
1	Agartala	IN021010100	23.88° N–91.25° E	16	79.88
2	Ahmedabad	IN005010600	23.07° N–72.63° E	55	80.57
3	Amritsar	INM00042071	31.71° N–74.80° E	230.4	70.28
4	Balasore	IN017010300	21.52° N–86.93° E	20	78.17
5	Bangalore	IN009010100	12.97° N–77.58° E	921	85.2
6	Begumpet Observatory	IN001080500	17.45° N–78.47° E	527	83.74
7	Bhopal Bairagarh	IN011351500	23.28° N–77.35° E	523	82.11
8	Bhuj Rudramata	IN005120501	23.25° N–69.67° E	80	76.63
9	Bikaner	IN019070100	28.00° N–73.30° E	224	70.35
10	Santacruz Mumbai	IN012070800	19.12° N–72.85° E	14	87.24
11	Chitradurga	IN009070100	14.23° N–76.43° E	733	80.45
12	Cuddalore	IN020020300	11.77° N–79.77° E	12	80.65
13	Dehradun	IN023160900	30.32° N–78.03° E	682	58.28
14	Gadag	IN009090300	15.42° N–75.63° E	650	79.23
15	Goa Panjim	IN022030600	15.48° N–73.82° E	60	84.39
16	Gorakhpur	IN023261300	26.75° N–83.37° E	77	56.14
17	Hissar	IN006031000	29.17° N–75.73° E	221	75.73
18	Indore	IN011170400	22.72° N–75.80° E	567	81.87
19	Jabalpur	IN011180800	23.20° N–79.95° E	393	73.46
20	Jaipur Sanganer	IN019131301	26.82° N–75.80° E	390	80.81
21	Jodhpur	IN019180500	26.30° N–73.02° E	224	72.48
22	Kakinada	IN001050200	16.95° N–82.23° E	8	81.46
23	Kozhikode	IN010050700	11.25° N–75.78° E	5	71.87
24	Kurnool	IN001120100	15.80° N–78.07° E	281	69.43
25	Lucknow Amausi	IN023351400	26.75° N–80.88° E	128	80.65
26	Machilipatnam	IN001111200	16.20° N–81.15° E	3	83.74
27	Madras Minambakkam	IN020040900	13.00° N–80.18° E	16	83.98
28	Mangalore Bajpe	IN009130300	12.92° N–74.88° E	102	87.97
29	Nellore	IN001160200	14.45° N–79.98° E	20	80.24
30	New Delhi Safdarjun	IN022021900	28.58° N–77.20° E	216	81.79
31	Patiala	IN018103100	30.33° N–76.47° E	251	76.46
32	Pbo Anantapur	IN001020700	14.58° N–77.63° E	364	80.98
33	Pendra Road	IN011060800	22.77° N–81.90° E	625	70.69
34	Poona	IN012190100	18.53° N–73.85° E	559	85.28
35	Raipur	IN011291000	21.22° N–81.67° E	294	38.58
36	Rajkot	IN005150100	22.30° N–70.78° E	138	80.41
37	Ratnagiri	IN012201100	16.98° N–73.33° E	67	86.91
38	Satna	IN011340100	24.57° N–80.83° E	317	55.2
39	Solapur	IN012230300	17.67° N–75.90° E	479	79.84
40	Srinagar	IN008010200	34.08° N–74.83° E	1587	64.27
41	Surat	IN005171200	21.20° N–72.83° E	12	80.81
42	Tezpur	IN003020100	26.62° N–92.78° E	79	56.54
43	Thiruvananthapuram	IN010100400	8.48° N–76.95° E	64	87.28
44	Tiruchchirapalli	IN020130700	10.77° N–78.72° E	88	80.89

**Table 2:** Characteristics of reports and cases of human fascioliasis in India. n.s. = not specified; d = days; m = months; y = years.

Origin of Reports and Cases:	No.	States with Diagnosed Cases:	No.
- Reports of cases (in 35 articles + 3 requests to the WHO)	59	- Northeastern India—state(s) n.s.	15
- Individual fascioliasis patients (–4 repeatedly reported)	55	- Uttar Pradesh	7
- Cases in which the locality of the patient was given	11	- Gujarat	5
- Cases including only the locality of the hospital	44	- West Bengal	5
- Cases imported from other countries	3	- Maharashtra	5
		- Punjab	3
**Specific diagnosis of causal agent:**		- Tamil Nadu	3
- Infected by *Fasciola hepatica*	31	- Jammu and Kashmir	2
- Infected by *Fasciola gigantica*	6	- Rajasthan	2
- Infected by admixed *Fasciola* hybrid	1	- Haryana	1
- Without specific diagnosis	17	- Delhi	1
		- Bihar	1
**Altitude of patient’s locality or hospital’s locality:**		- Jharkhand	1
- Between 1 and 500 m a.s.l.	35	- Assam	1
- Between 500 and 600 m a.s.l.	2	- Arunachal Pradesh	1
- Higher than 1000 m a.s.l.	2	- Madhya Pradesh	1
- Unknown because of lack of patient’s or hospital’s locality	16	- Odisha	1
		- State n.s. in traveler	1
**Sex and age of patient:**			
- Male/female	21/33	**Drugs used for patient’s treatment:**	
- Sex n.s.	1	- Triclabendazole	30
- Age—extreme values	30 m–71 y	- Albendazole	10
- Age—mean (in years)	33.1	- Nitazoxanide	21
- Age—mean for males/for females (in years)	33.4/32.7	- Diethylcarbamazine	1
		- Praziquantel	4
**Time elapsed between symptom onset and diagnosis:**		- Antibiotics plus steroids	2
- Extreme values	10 d–5 y	- Bitionol	1
- Mean	9.2 m	- Ivermectine	6
**Diagnostic method:**		**Coinfecting parasites:**	
- By egg finding (in stool, or duodenal or bile aspirate)	12	- *Giardia intestinalis*	1
- By adult finding (in ERCP, surgery, or liver biopsy)	28	- *Hymenolepis nana*	1
- By serology	3	- *Ascaris lumbricoides*	1
- By clinics and image techniques (US, CT, MRCP)	12	- *Trichuris trichiura*	1

**Table 3:** The main, secondary, tertiary, and quaternary routes, and respective main nodes, used by human-guided livestock movements for the transport of humans and goods, including from long, mid- and short distances to seasonal horizontal or vertical transhumance and nomadic pastoralism.

Routes	Category	Main Nodes (see Map in Figure 8)	Starting Period	Purpose/Use	References
Grand Trunk Road (= Uttarapatha; = northern route)	Main route	Kophen, Peshawar, Taxila, Gandhara, Lahore, Indraprastha, Mathura, Sravasti, Prayagraj, Kushinagar, Pataliputra, Chandraketugarh, Tamralipti, Teknaf	500 y BC	Long-distance goods transport	[1,75]
Daksinapatha Road (= southern route)	Main route	– Main route: Rajgrahi, Varanasi, Bharhut, Vidisa, Ujjayini, Ajanta, Pratisthana – Connections with the Grand Trunk Road: Kausambi, Gwalior, Mandasor, Bairat. – Connections with seaports of the Maritime Silk Road: Sanjeli, Nasik, Bhorgat, Bhaja	500 y BC	Long-distance goods transport	[75]
Silk Road	Main route	Taxila–Srinagar–northern Silk Road; Sravasti–Khotan in northern Silk Road	2500–2000 y BC	Long-distance goods transport	[1,76]
Tea-Horse Road	Main route	Sikkim state–Lhasa–Yaan; Dhaka–Dali; Mandalay–Dali	700 y BC	Long-distance goods transport	[1]
Maritime Silk Road	Main route	– West Indian coast: Barbarikon, Hayhab, Gogha, Khambhat, Barygaza, Kamrej, Nala Sopera, Mumbai, Kalyan, Chaul, Goa, Muziris – East Indian coast: Madras, Machilipatnam	200 y BC	Long-distance goods transport, seaport to hinterland	[1,75]
Sea–Grand Trunk Road connection	Secondary route	Barbaricon–Multan/Indus Valley–Taxila	300 y BC– 100 y AD	Mid-distance goods transport	[1,75,77]
Orissa–Bangladesh connection	Secondary route	Bhubaneswar–eastern end of Grand Trunk Road and Tea-Horse Road	200 y BC	Long-distance goods transport	[78]
Arabian Sea– hinterland connections	Tertiary route	– Barygaza (and neighboring seaports) – Sanjeli, Mandasor, Ujjayini – Nala Sopara/Mumbai, Kalyan, Nasik, Pratisthana – Chaul, Bhorgat, Bhaja, Pratisthana	500 y BC	Short-distance goods transport	[1,75]
Bay of Bengal–Daksinapatha	Tertiary route	Madras–Pratisthana; Machilipatnam–Pratisthana	1500 y AD	Mid-distance goods transport	[75]
Gujarat intra-/inter-state movements	Quaternary route	Gujarat state lowlands		Horizontal lowland seasonal transhumance	[79,80,81]
Rajasthan intra-/interstate movements	Quaternary route	Rajasthan state lowlands		Horizontal lowland seasonal transhumance	[79,80]
Madhya Pradesh intra-/interstate movements	Quaternary route	Madhya Pradesh state lowlands		Horizontal lowland seasonal transhumance	[79,80]
Uttar Pradesh Intra-/interstate movements	Quaternary route	Uttar Pradesh state lowlands		Horizontal lowland seasonal transhumance	[79,82]
Tamil Nadu intra-/interstate movements	Quaternary route	Tamil Nadu state lowlands		Horizontal lowland seasonal transhumance	[79]
Punjab intra-/inter- state movements	Quaternary route	Punjab state lowlands		Horizontal lowland seasonal transhumance	[79]
Western Himalaya vertical movements	Quaternary route	Jammu and Kashmir–Himachal Pradesh–Uttarakhand		Vertical altitudinal seasonal transhumance	[79,80,82,83,84,85,86,87]
Central Himalaya vertical movements	Quaternary route	Eastern Nepal/Sikkim–Lhasa/Bhutan		Vertical altitudinal seasonal transhumance	[88,89,90,91,92]
Eastern Himalaya vertical movements	Quaternary route	Arunachal Pradesh–Tea-Horse Road		Vertical altitudinal seasonal transhumance	[93,94,95,96,97]
Eastern states	Quaternary routes	Bihar, Jharkhand, Orissa, Chhattisgarh, and West Bengal		Nomadic pastoralism	[98,99,100,101]

**Table 4:** States and union territories of India and their transhumant, nomadic, or pastoralist groups moving livestock reservoir species, and respective reported liver fluke infection prevalences. Summarized data from various sources. n.d. = no data.

States of India	State Subzones	Transhumant, Nomadic, or Pastoralist Groups	Moving Livestock Reservoir Species	Ruminant Prevalences Reported
Andhra Pradesh		Golla	Cattle	Cattle & buffaloes: 3.8%
		Kuruma	Sheep	
Arunachal Pradesh	Tawang and Kemeng districts	Monpa	Cattle, yak	Mithun: 4.08–28.9%
Assam				Cattle: 34.0%
Bihar		Gaddi Muslim/Ghosi	Cattle	Sheep: 8.98%; goats: 12.87%
Chhattisgarh				Cattle: 0.3%; buffaloes: 0.6%
Delhi				Sheep, goats, cattle, buffaloes: 0.8–2.1%
Goa		Gavli	Cattle	n.d.
Gujarat		Bharwad	Sheep, goats, cattle	Cattle & buffaloes: 12.0–42.0% Buffaloes: 25.0–33.0%
	Gir forest region	Charan	Cattle	
		Gavli	Cattle	
	Kutch region	Jath	Cattle, camelids	
	Saurashtra region	Mer	Cattle, camelids	
		Rebari/Reika	Goats, cattle, camelids	
Haryana		Gaderia	Sheep, goats	Buffaloes: 1.3%
Himachal Pradesh		Gaddis	Sheep, goats	Sheep & goats: 8.8–9.6% Cattle & buffaloes: 36.0–48.7%
		Gujjar	Buffalo, cattle	
	Kinnaur district	Kinnaura	Sheep, goats	
Jharkhand		Pashu Sakhis	Mostly goats	n.d.
Jammu and Kashmir		Bakarwal	goats	Sheep: 15.0% Sheep & goats: 2.0–4.2% Cattle: 4.5–85.0% Cattle & buffaloes: 2.6–6.9%
	Zanskar	Changpa	Yak	
		Gaddis	Sheep, goats	
		Gujjar	Buffalo, cattle	
Karnataka		Dhangar/Kuruba	Sheep	Cattle: 2.4–26.6% Buffaloes: 1.5–22.5%
		Gavli/Toda	Cattle	
Kerala		Toda	Cattle	n.d.
Madhya Pradesh		Dhangar	Sheep	Goats: 3.5–25.3%; cattle: 6.02%; buffaloes: 3.51%
		Gaderia	Sheep, goats	
Maharashtra		Dhangar	Sheep	Sheep: 31.8%; goats: 19.4%; cattle: 17.5%; buffaloes: 23.8%
		Gavli/Golla	Cattle	
Manipur		Farmers	Goats, cattle	n.d.
Meghalaya		Farmers, Scheduled Tribe	Sheep, goats, cattle, buffaloes	Cattle: 52.2%
Mizoram		Farmers	Cattle	n.d.
Nagaland		Farmers	Sheep, cattle, buffaloes	n.d.
Orissa (= Odisha)				Cattle: 4.3%
Punjab		Banjaras	Cattle	Sheep: 72.9%; goats: 56.8% Cattle: 1.9–18.0%; buffaloes: 21.0%
Rajasthan		Gaddi Muslim/Ghosi	Cattle	Cattle: 2.4%
	Mewar in the south	Gayri	Sheep, cattle	
		Gujjar	Buffalo, cattle	
	Ganganagar and Bikaner districts	Rath	Cattle	
		Rebari/Reika	Goats, cattle, camelids	
	Marwar region	Sindhi	Sheep, cattle, camelids	
	Jaisalmer	Sindhi	Sheep, cattle, camelids	
Sikkim	North district	Bhutia	Sheep, goats, cattle	Goat & cattle: 19.3–83.0%
Tamil Nadu	Nilgiri region	Toda	Cattle	Sheep: 50.0%
Telangana	Mahabubnagar dist.	Farmers	Sheep, goats, cattle, buffaloes	n.d.
Tripura		Farmers	Sheep, goats, cattle, buffaloes	n.d.
Uttar Pradesh		Gaddi Muslim	Cattle	Sheep: 1.7% Goats: 2.0–3.6% Buffaloes: 32.8–94.0%
		Gaderia	Sheep, goats	
		Ghosi	Cattle	
		Van Gujar	Buffalo	
Uttarakhand	Garhwal, Kumaon	Bhotia	Sheep, goats, cattle	Sheep: 2.5–4.5%; goats: 1.2–3.5%; buffaloes: 57.4–78.0%
		Van Gujar	Buffalo	
West Bengal				Cattle: 0.8%

## Data Availability

Data supporting the conclusions of this article are included within the article.

## Abbreviations

The following abbreviations are used in this manuscript:

a.s.l.	Above sea level
BC	Before Christ
DF	Number of days with frost
DP	Number of days with precipitation
ELISA	Enzyme-linked immunosorbent assay
EMT	Extreme maximum temperature
EmT	Extreme minimum temperature
epg	Eggs per gram of stool
ERCP	Endoscopic retrograde cholangiopancreatography
ETD	Extreme temperature difference
GDD	Growing degree-days
MET	Mean environmental temperature
MMT	Mean maximum temperature
MmT	Mean minimum temperature
MTD	Maximum temperature difference
mtDNA	Mitochondrial DNA
NTD	Neglected tropical diseases
PET	Total potential evapotranspiration
Prcp	Precipitation
rDNA	Ribosomal DNA
Wb-bs index	Water budget-based system index
